# Drought resistance is mediated by divergent strategies in closely related Brassicaceae

**DOI:** 10.1111/nph.15841

**Published:** 2019-06-18

**Authors:** Nora Marín‐de la Rosa, Chung‐Wen Lin, Yang Jae Kang, Stijn Dhondt, Nathalie Gonzalez, Dirk Inzé, Pascal Falter‐Braun

**Affiliations:** ^1^ Institute of Network Biology (INET) Helmholtz Zentrum München (HMGU) München‐Neuherberg 85764 Germany; ^2^ Division of Life Science Gyeongsang National University Jinju 52828 Korea; ^3^ Department of Plant Biotechnology and Bioinformatics Ghent University Ghent 9052 Belgium; ^4^ VIB‐UGent Center for Plant Systems Biology VIB Ghent 9052 Belgium; ^5^ UMR 1332 Biologie du Fruit et Pathologie INRA Univ. Bordeaux Villenave d'Ornon Cedex 33882 France; ^6^ Microbe–Host Interactions Ludwig‐Maximilians‐Universität (LMU) München Munich 80539 Germany

**Keywords:** Arabidopsis, Brassicaceae, comparative phenotyping, drought, high‐throughput phenotyping, stress resistance, systems biology, transcriptome

## Abstract

- Droughts cause severe crop losses worldwide and climate change is projected to increase their prevalence in the future. Similar to the situation for many crops, the reference plant *Arabidopsis thaliana* (*Ath*) is considered drought‐sensitive, whereas, as we demonstrate, its close relatives *Arabidopsis lyrata* (*Aly*) and *Eutrema salsugineum* (*Esa*) are drought‐resistant.
- To understand the molecular basis for this plasticity we conducted a deep phenotypic, biochemical and transcriptomic comparison using developmentally matched plants.
- We demonstrate that *Aly* responds most sensitively to decreasing water availability with early growth reduction, metabolic adaptations and signaling network rewiring. By contrast, *Esa* is in a constantly prepared mode as evidenced by high basal proline levels, ABA signaling transcripts and late growth responses. The stress‐sensitive *Ath* responds later than *Aly* and earlier than *Esa*, although its responses tend to be more extreme. All species detect water scarcity with similar sensitivity; response differences are encoded in downstream signaling and response networks. Moreover, several signaling genes expressed at higher basal levels in both *Aly* and *Esa* have been shown to increase water‐use efficiency and drought resistance when overexpressed in *Ath*.
- Our data demonstrate contrasting strategies of closely related Brassicaceae to achieve drought resistance.

## Introduction

Approximately 50% of annual crop yield losses are attributable to droughts (Boyer, 1982) and the frequency and severity of drought conditions are projected to worsen in coming years (Anderson‐Teixeira *et al*., 2013; Heffernan, 2013). As many elite cultivars tend to be drought‐sensitive, ensuring food security will require development of more drought‐resistant, high‐yield varieties. Importantly, most crops have wild relatives that are much more drought‐resistant, suggesting an evolutionary plasticity that holds biotechnological potential (Nevo & Chen, 2010; Zhang *et al*., 2017). Understanding the molecular basis of differential drought sensitivity in closely related species is therefore expected to aid crop improvement.

Drought stress resistance is a complex phenotype resulting from the interplay of many traits, each of which is regulated by numerous, often pleiotropic genes that determine cell‐type‐specific molecular networks. Here the concept of ‘phenes’ will be useful (Porter, 1973; Lynch *et al*., 2014), which denotes low‐level phenotypic traits for which in principle the molecular mechanisms and underlying networks can be delineated, for example cell division. Once the manifestation of phenes affecting a complex trait can be described quantitatively, it may be possible to model the higher level phenotype as the combinatorial interaction of all phenes. For this, however, detailed knowledge on the phenes mediating drought resistance in different species is required.

As a consequence of this complexity, different drought resistance strategies exist, which differ in the dominant phenes (Turner, 1986; Aguirrezabal *et al*., 2006; Yang *et al*., 2010). During drought *escape*, plants trigger mechanisms to accelerate completion of their life cycle, set seed and thus secure the next generation (Fleury *et al*., 2010). During drought *avoidance*, plants reduce water loss to maintain tissue water content. Lastly, drought *tolerance* is characterized by osmotic adjustments and protection of cells from damage due to desiccation and high osmolarity (Tardieu, 2013). While the metabolic and some signaling pathways involved in the individual strategies have been characterized, a systems understanding and the respective pathway integration and decision points remain elusive. Detailed comparative phenotypic data are required that can form the basis of mechanistic studies.

Similar to the situation in many crops, the reference plant *Arabidopsis thaliana* (*Ath*) is considered sensitive to drought and salt stress. The closely related *Eutrema salsugineum* (*Esa*) exhibits a much higher tolerance to salt and water deprivation, and has been proposed as an extremophile model to investigate mechanisms underlying resistance to drought, salinity and freezing (Inan *et al*., 2004; Taji *et al*., 2004; Wong *et al*., 2006; Griffith *et al*., 2007; Higashi *et al*., 2013; Xu *et al*., 2014). *Arabidopsis lyrata* (*Aly*) is a closer *Ath* relative and has been described as resistant to freezing and drought (Sletvold & Agren, 2011; Wos & Willi, 2018). *Aly* and *Esa* display high morphological, developmental and metabolic similarities with *Ath* (Amtmann, 2009; Hu *et al*., 2011; Yang *et al*., 2013). *Ath* and its relatives constitute an excellent system to elucidate the molecular mechanisms and evolutionary adjustments underlying drought resistance in closely related species. We aimed to understand which molecular changes contribute to this phenotypic plasticity within Brassicaceae.

## Materials and Methods

### Plant material

*Ath* (Col‐0 ecotype) was obtained from Nottingham Arabidopsis Stock Center (http://arabidopsis.info). *Aly* strain MN47 (Hu *et al*., 2011) and *Esa* (accession Shandong) (Yang *et al*., 2013) were kindly provided by Juliette de Meaux (University of Cologne) and Erich Glawischnig (Technische Universität München).

### Plant growth conditions and drought treatment

The plant phenotyping platform (WIWAMxy) at VIB Ghent (www.wiwam.com) was used for high‐throughput phenotypic characterization. Pots were prepared as described (Skirycz *et al*., 2011b). Briefly, all pots (128 per species) were radio frequency identification (RFID)‐tagged and the dry soil weight of individual pots was calculated. Three to four plants were sown after 4 d of stratification at 4°C in the dark. Pots were placed on WIWAMxy and covered with plastic film for 3 d to maintain humidity. On day 4 the cover was removed and the well‐watered condition (WW) of 2.19 g water g^−1^ soil was maintained robotically. When two complete open cotyledons were observed in all pots, one average‐sized seedling per pot was kept. Daily, images of the plants were taken, each pot was weighted, positions were randomized and water was added to precisely maintain WW conditions.

Water deficit (WD) treatment started when leaf 6 (L6) was initiated on the apex (1 mm, developmental stage 1.06) as judged by manual inspection (Boyes *et al*., 2001). Watering for the WD group (78 pots per species) was stopped at 14 (*Ath*), 20 (*Esa*) and 22 (*Aly*) d after sowing (DAS). After 15 d without watering, plants were rewatered and survival was scored 3 d later. Two independent replicates were performed in trays. Plants were grown under constant environmental conditions: 16 h day, 21°C, 55% relative humidity and 110–120 μmol m^−2^ s^−1^ light intensity.

### Growth measurements

For analysis, visualization and management of phenotypic datasets, the PSB Interface for Plant Phenotype Analysis (https://pippa.psb.ugent.be) was used. Segmented images were used to measure projected rosette area, perimeter and convex hull area to calculate relative growth rate (RGR), stockiness and compactness.

$$\text{RGR}=\frac{\ln\left(A_{t}\right)-\ln\left(A_{t-\Delta t}\right)}{\Delta t},$$

$$\text{Stockiness}=4\frac{\pi \times \text{area}}{{\text{perimeter}}^{2}},$$

$$\text{Compactness}=\frac{\text{Rosette area}}{\text{Convex hull area}}.$$

### Individual leaf area and cellular analysis

For analysis of individual leaf growth, 10 plants per species and treatment were harvested at 25 (*Ath*), 33 (*Aly*) and 31 (*Esa*) DAS and photographed. Individual leaf area was calculated using imagej (https://imagej.nih.gov/ij/). For cellular analysis, Chl of leaf 6 was removed and five to eight leaves were used for cellular drawings and analysis as described (Andriankaja *et al*., 2012). After calibration, cells numbers per leaf were calculated as the product of total leaf area and the average cell number per area. Stomatal index (SI) was calculated as:

$$\text{SI}=\frac{\text{Number of guard cells}}{\text{Number of epidermal cells}}.$$

### Stomatal aperture

Rosette leaves of 4‐wk‐old plants were incubated under light conditions for 3 h with buffer (10 mM KCl, 10 mM CaCl_2_ and 10 mM MES, pH 6.5) with or without 10 μM ABA (Sigma Aldrich). The ratio between the width and length of ostiols (*R*_wl_) was measured.

### Measurement of maximum efficiency of photosystem II (*F*_v_/*F*_m_)

Chl fluorescence measurements were carried out employing the IMAGING‐PAM Chlorophyll Fluorescence System and imagingwin software (Heinz Walz, Effeltrich, Germany). *F*_v_/*F*_m_ measurements were obtained by application of a single saturating pulse to dark‐adapted plants. Average *F*_v_/*F*_m_ of the entire rosette was calculated using the imagej macro ‘PHENOPSIS‐Fluo’ (Bresson *et al*., 2015).

### Proline and anthocyanin measurement

Total rosettes were collected, and fresh weight was measured before freezing in liquid nitrogen. Proline content was determined spectrophotometrically using ninhydrin (Shabnam *et al*., 2016). Briefly, *c*. 50 mg of plant material was homogenized with 0.4 ml of 70% ethanol, and centrifuged for 5 min at 13 800 ***g***. Then, 50 μl extracts were incubated with 100 μl reaction mix (ninhydrin 1% (w/v); acetic acid 60% (v/v); ethanol 20% (v/v)) for 20 min at 95°C. Absorbance at 520 nm was measured for 100 μl of the reaction in a microplate reader. Anthocyanins were extracted with five volumes of extraction buffer (45% methanol; 5% acetic acid) for 5 min (Gechev *et al*., 2013). Extracts were centrifuged twice for 5 min at 13 800 ***g***; relative anthocyanins levels are reported as (*A*_530_ − *A*_657_) per g FW. Three independent experiments were performed with similar results (not shown).

### RNA extraction and sequencing

Total rosettes at days 0, 5, 11 and 14 after watering stop were collected and RNA was extracted with TRIzol (Invitrogen) according to the manufacturer's protocol. RNA was subjected to DNA digestion with RQ1‐RNase‐Free‐DNase (Promega). Impurities were removed with an RNeasy clean‐up‐kit (Qiagen). Libraries were prepared using the TruSeq RNA Sample Preparation Kit v. 2 (Illumina, San Diego, CA, USA). Sequencing was done using a HiSeq2500 with the HiSeq SBS Kit_v4 (Illumina) in paired‐end mode with a read length of 100 bp. Each experiment was performed with three biological replicates. RNA‐sequencing (RNA‐seq) data were deposited at NCBI (SRP155798). Adapter removal and quality‐based sequence trimming data was done with trimmomatic v.0.36 (Bolger *et al*., 2014). fastqc (http://www.bioinformatics.babraham.ac.uk/projects/fastqc) was used for read quality control before and after trimming. High‐quality reads were mapped to the *Ath* (TAIR10), *Aly* (v.2.1) and *Esa* (v.1.0) reference genomes and quantified using kallisto (Bray *et al*., 2016). TPM (transcripts per million) values for genes were generated by summing TPM values for the corresponding transcripts generated by a custom Perl script. Genes with at least one sample with a log_2_TPM ≥ 1 were used for downstream analysis.

### RNA‐seq analysis

Orthology relationships among *Ath*,* Aly* and *Esa* were identified using blastp with a 10^−3^ *E*‐value cutoff. Coexpressed gene modules were identified using a weighted gene co‐expression network analysis (WGCNA) (Langfelder & Horvath, 2008). A matrix of pairwise correlations between all pairs of genes across all samples was constructed and raised to a soft‐thresholding power (β = 16). Modules of coexpressed genes were identified by calculating topological overlap (TOM)‐based dissimilarity, which was used as input to average linkage hierarchical clustering. Submitting the resulting dendrogram to a dynamic tree‐cutting algorithm and merging threshold function at 0.1, we identified 28 modules. Each module was identified by its Eigengene calculated as the first principal component of the gene expression pattern. The topgo and limma packages (Alexa *et al*., 2006; Diboun *et al*., 2006) were used to identify enriched Gene Ontology (GO) and Kyoto Encyclopedia Genes and Genomes (KEGG) pathway annotations. The GO annotation dataset (ATH_GO_GOSLIM) was obtained from TAIR (http://www.arabidopsis.org). Biological function analysis was performed using a WGCNA in combination with Fisher's exact test included in the topgo package, from which GO enrichment was determined using revigo (Supek *et al*., 2011). Multiple testing correction was estimated via false discovery rate (FDR).

Significance of overlap of common regulated expressed mutual orthologs (EMOs) between *Ath*,* Aly* and *Esa* was estimated using a 1000‐fold permutation simulation. The respective same number of drought‐regulated EMOs was randomly selected from all common orthologs and the overlap was determined. The experimental *P*‐value was calculated by dividing the number of samplings in which the number of random selected targets was greater than or equal to the observed number of common regulated genes by the number of samplings performed. If the observed value of common regulated genes was not seen in the simulation, the *P*‐value was set to < 0.001.

## Results

### Analyzing drought response in growth‐stage‐synchronized plants

**Figure 1:**
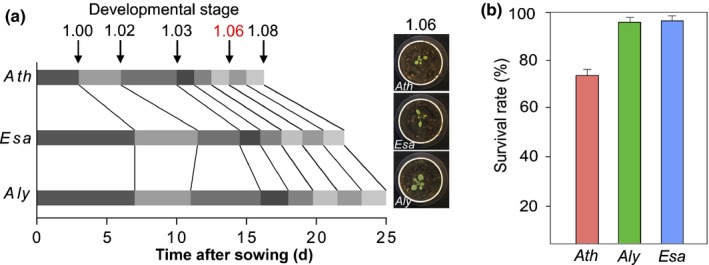
Growth stage progression and drought resistance in *Arabidopsis thaliana* (*Ath*), *Arabidopsis lyrata* (*Aly*) and *Eutrema salsugineum* (*Esa*). (a) Scheme of chronological progression of *Ath*,* Aly* and *Esa*. Boxes represent the time between subsequent developmental stages. Red label (1.06) indicates the start of the water deficit period (T0). Representative pictures of plants at developmental stage 1.06 are shown. (b) Survival rates of the three species after rewatering. Data are represented as mean of three independent replicates ± SD (*n *=* *27 per replicate).

As an important first step towards understanding drought stress responses among Brassicaceae we conducted a controlled comparative study of the drought‐sensitive *Ath* and its reportedly more resistant relatives *Aly* and *Esa*. Critical for comparative drought studies is the dependence of water requirement on developmental parameters (Xu *et al*., 2009; Skirycz *et al*., 2010; Verelst *et al*., 2010; Negrao *et al*., 2017). As developmental timing differs between the species, we first defined the developmental progression of soil‐grown *Ath*,* Aly* and *Esa* plants (Boyes *et al*., 2001). In WW conditions *Ath* followed the previously described timeline (Boyes *et al*., 2001), while leaf emergence was slower in *Aly* and *Esa* (Fig. 1a). This difference was most pronounced from germination to the emergence of the third leaf (stage 1.03) and was more synchronized subsequently (Fig. 1a). For physiological and developmental comparability, plants at stage 1.06 were used as a starting point, which *Ath* reached at 14 DAS, *Aly* at 22 DAS and *Esa* at 22 DAS. For each species 128 plants were grown, watered and imaged using the WIWAMxy phenotyping platform (Skirycz *et al*., 2011b). At developmental stage 1.06 (T0) watering was stopped for 15 consecutive days (T0–T14) for the WD subset. At T14 WD plants presented visual signs of wilting and were rewatered to determine survival rate as a measure of drought resistance. Nearly all *Aly* and *Esa* individuals recovered, corresponding to survival rates of 96% and 98%, respectively; only 76% of *Ath* plants survived the severe drought period (Fig. 1b). The differential survival was mirrored by the maximum efficiency of photosystem II (*F*_v_/*F*_m_) (Woo *et al*., 2008), which fell below *c*. 0.65 for predominantly those individuals that did not recover after rewatering (Supporting Information Fig. S1). These observations confirmed previous reports describing an increased *Esa* drought resistance (Xu *et al*., 2014). Interestingly, *Aly* and *Esa* showed essentially the same level of drought resistance. At the same time the difference between *Esa* and *Ath* was less pronounced than we had expected based on prior reports (Ghars *et al*., 2008; Yu & Li, 2014), suggesting that previously described ‘resistance’ partly resulted from developmental differences. Nonetheless, the clear drought resistance differences between *Ath* and both *Aly* and *Esa* forms the basis for elucidating the underlying physiological response phenes and molecular mechanisms.

### Rosette growth dynamics are affected by drought stress

**Figure 2:**
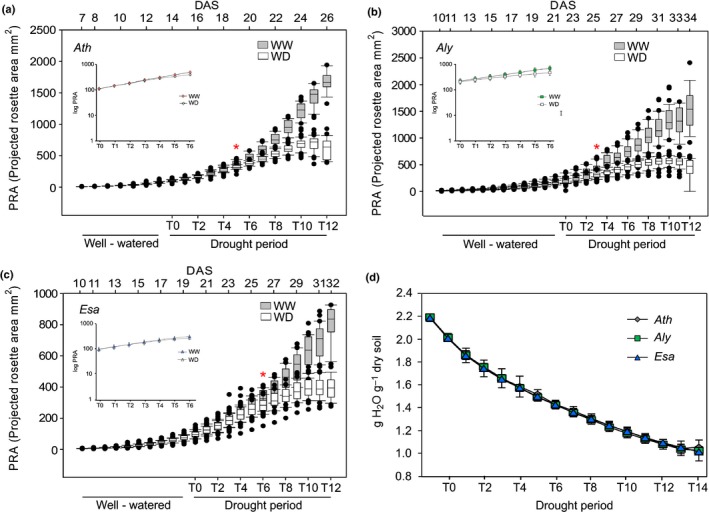
Rosette growth dynamics under well‐watered (WW) and water deficit (WD) conditions. Projected rosette area (PRA) over time of (a) *Arabidopsis thaliana* (*Ath*), (b) *Arabidopsis lyrata* (*Aly*) and (c) *Eutrema salsugineum* (*Esa*). Asterisks indicate the first day with a significant reduction of growth of WD plants compared to respective WW controls (*P* ≤ 0.05, Student's *t*‐test). *n *>* *25 plants per time point and treatment. Inset: for better visualization of early time points PRA is represented on log scale (from T0 to T12). (d) Soil water content for all three species from T0 to T14 (*n *=* *25 plants per time point; data are represented as mean ± SD). Numeric data are provided in Supporting Information Table S1.

As growth reduction is one of the earliest plant responses to drought (Aguirrezabal *et al*., 2006; Pereyra‐Irujo *et al*., 2008; Skirycz & Inze, 2010; Tardieu *et al*., 2010; Baerenfaller *et al*., 2012), we determined growth over time from the projected rosette area (PRA). Although all three species responded to decreasing water availability with reduced rosette growth, their dynamics differed profoundly. *Aly* responded first to water deprivation at T3, whereas *Ath* and *Esa* showed significant growth reduction only at T5 and T6, respectively (inset Fig. 2a–c). Notably, on the first day of treatment (T0) *Aly* PRA (211 mm^2^) was considerably larger than those of *Esa* (94 mm^2^) and *Ath* (100 mm^2^) (Table S1). However, the larger *Aly* rosette did not result in higher water consumption after watering‐stop (Fig. 2d), which could be a reason for the faster growth reduction. The measured soil water content at the respective time of growth reduction was 1.76 (*Aly*), 1.66 (*Ath*) and 1.50 (*Esa*) g water g^−1^ dry soil (Fig. 2d; Table S1). Thus, while expectedly all three species responded with growth reduction to reduced water availability, remarkably the two resistant species showed opposite response dynamics relative to the sensitive *Ath*. This differing response could indicate that despite their evolutionary proximity *Aly* and *Esa* evolved different strategies towards stress resistance.

Following growth reduction, *Ath* and *Aly* entered a short adaptation period, which was not observed in *Esa* (Fig. S2a–c), but has been reported for *Ath* following osmotic stress (Skirycz *et al*., 2011a). In contrast to reports for other ecotypes and treatments (Jansen *et al*., 2009; Dhondt *et al*., 2014), morphological rosette parameters exhibited no response differences (Fig. S3a,b).

Thus, contrary to our expectation based on the enhanced survival of *Aly* and *Esa* and their evolutionary proximity, our phenotypic analysis revealed dramatic response differences of the growth phene among the two resistant species.

### Leaf growth in response to drought stress

**Figure 3:**
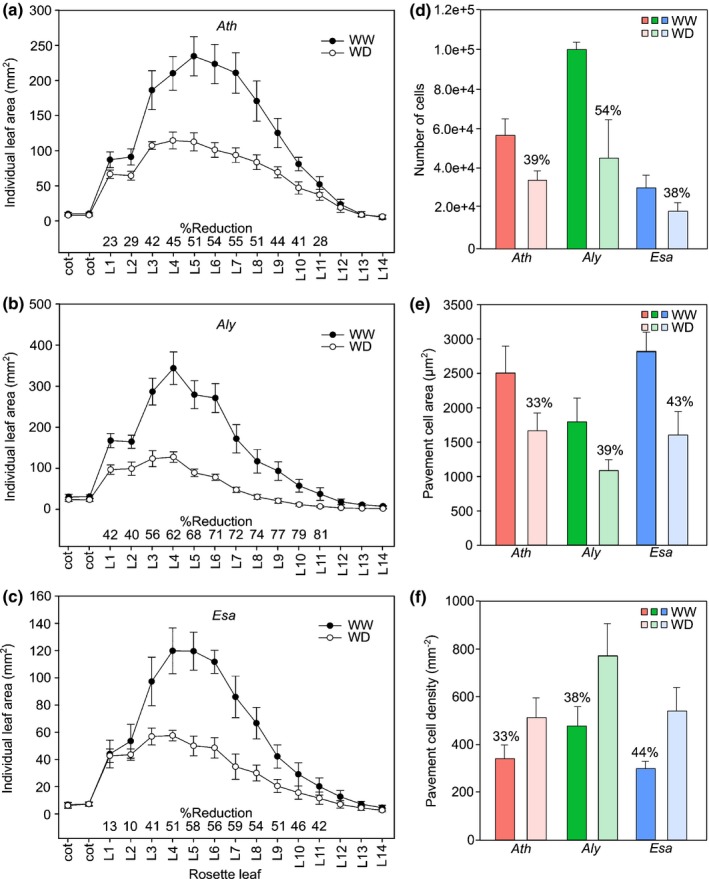
Leaf and cellular parameters of well‐watered (WW) and water deficit (WD) plants. Average area of detached leaves of (a) *Arabidopsis thaliana* (*Ath*), (b) *Arabidopsis lyrata* (*Aly*) and (c) *Eutrema salsugineum* (*Esa*). The *x*‐axis represents cotyledons (cot) and individual leaves in order of appearance in the rosette (L1–L14). Inset ‘% reduction’ indicates relative decrease of WD relative to WW leaf sizes (*n *=* *10 plants per species and treatment; data are represented as mean ± SD). Cellular characteristics of leaf 6 (L6) are calculated from microscopic drawings of the abaxial leaf epidermis. (d) Estimated cell number, (e) average pavement cell area and (f) pavement cell density (*n *=* *5–8 plants per species and treatment; data are represented as mean ± SD). Numeric data are provided in Supporting Information Table S1.

To better understand the basis of growth reduction we analyzed leaf size in more detail. In WD conditions leaf area of all species (L1–L11), except cotyledons and late emerging leaves, showed a dramatic decrease (Fig. 3a–c). This growth reduction was most prominent in *Aly* (60%), whereas in *Ath* and *Esa* the respective reductions of 42% and 44% were comparable (Fig. S4). Of note is the rapid strong response of *Aly* leaves, exemplified by a 42% surface area reduction of *Aly* L1, in contrast to 13% and 23% reduction of L1 in *Esa* and *Ath*, respectively. Cellular analysis of mature L6 revealed that cell size and cell number are reduced to a similar extent in *Ath*. In *Aly*, cell number (i.e. proliferation) was most drastically reduced whereas in *Esa,* cell size (i.e. growth) was most prominently affected (Fig. 3d,e). The reduction in cell area and number led to a higher cell density in all species (Fig. 3f), whereas the stomatal index was only minutely reduced by drought (Fig. S5a). Stomatal area was similarly reduced upon drought in all three species, while the increase of stomatal density was more prominent in *Aly* plants (Fig. S5b,c). These observations add to the evidence that *Aly* and *Esa* display different drought response phenes.

These phenotypic analyses revealed substantial differences in the specific drought responses of closely related Brassicaceae. Most remarkable is the contrasting behavior of the drought‐resistant *Aly* and *Esa* in several response phenes. While *Aly* responds most sensitively to drought stress, the similarly resistant *Esa* responds much later with growth reduction and even later than the sensitive *Ath*. We aimed to understand the underlying molecular changes using transcriptional profiling.

### Transcriptome dynamics in response to drought stress

**Figure 4:**
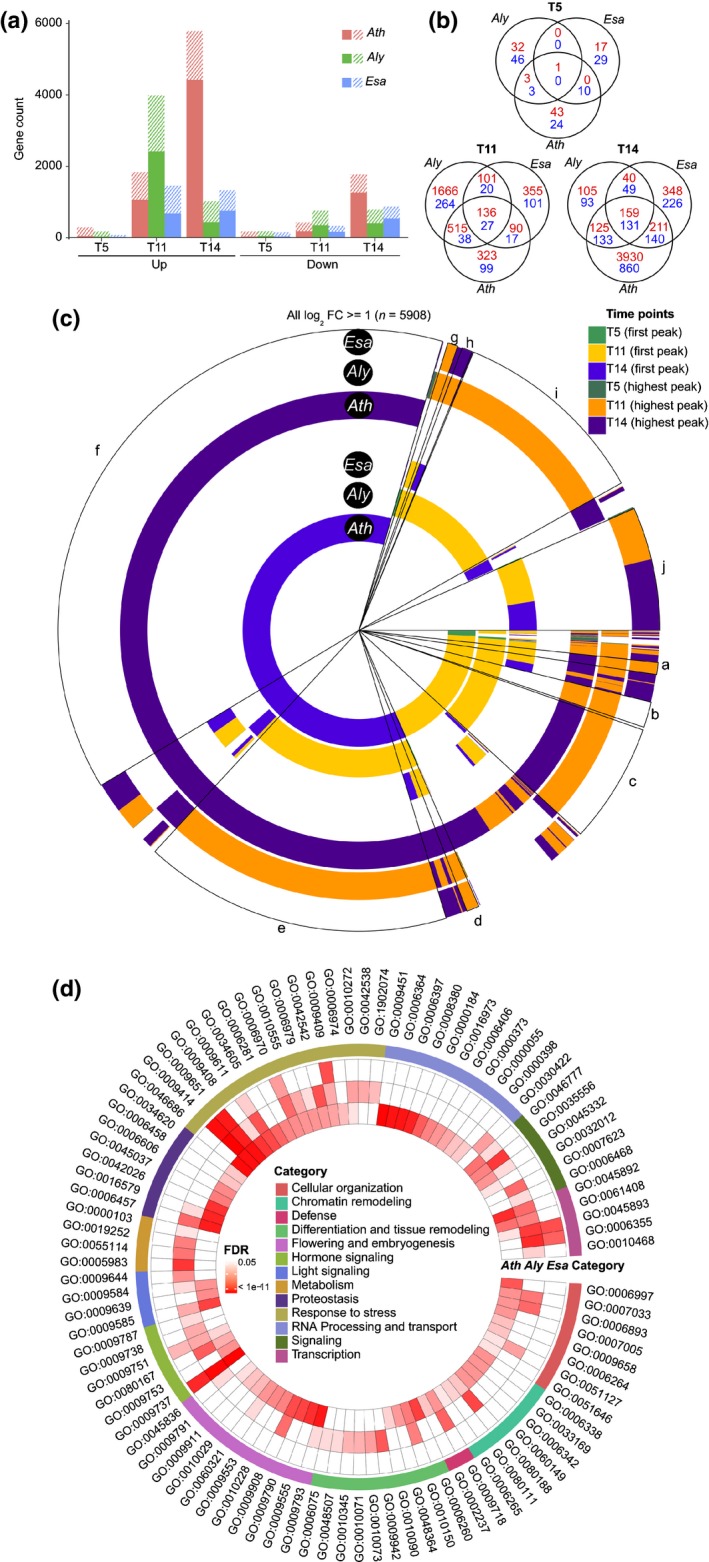
Comparative profiling of transcriptional drought stress responses. (a) Total number of differential expressed genes. Bars represent the number of differentially regulated (WD/WW) genes in *Arabidopsis thaliana* (*Ath*), *Arabidopsis lyrata* (*Aly*) and *Eutrema salsugineum* (*Esa*). Solid parts represent expressed mutual orthologs (EMOs), and shaded parts non‐EMOs. (b) Venn diagrams of commonly and specifically drought‐induced EMOs at T5, T11 and T14. Red indicates log_2_ fold change ≥ 1, while blue indicates log_2_ fold change ≤ −1. (c) Circle representation showing the first and highest peak of each drought‐induced EMO. (d) Circle diagram of GO terms enriched in upregulated genes (numeric Data in Supporting Information Table S5). WD, water deficit; WW, well‐watered.

To study the genome‐wide transcriptomic changes, we collected total rosettes at four time‐points (T0, T5, T11 and T14) of WW and WD plants for RNA‐seq‐based transcriptome analysis. The total number of expressed genes with a log_2_TPM > 1 was 20 586 (*Ath*), 21 092 (*Aly*) and 19 708 (*Esa*), representing 74.4%, 67.9% and 74.8% genome coverage, respectively. Of note is the different pattern of transcriptional changes between the three species. Whereas for *Aly* dramatic changes are evident at T11, *Ath* responses peak only at T14, but more genes are induced. By contrast, the transcriptional changes in *Esa* are rather moderate, indicating that this species may require fewer transcriptional adjustments (Fig. 4a; Tables S2, S3), possibly reflecting a more drought‐prepared state. Interestingly, in all species most genes that were upregulated in response to drought were already expressed before stress onset at T0. Only 328 (*Ath*), 149 (*Aly*) and 134 (*Esa*) genes are expressed specifically in response to drought stress (Fig. S6; Table S4) but these are enriched in ‘response to water deprivation’ functions (Fig. S7; Table S5).

At T5 only a few transcriptional changes can be observed in all three species, suggesting that the initial physiological responses, for example growth reduction, are mediated predominantly by post‐translational mechanisms. In *Aly* was the drought stress marker RD29B was upregulated at T5, whereas in *Ath* and *Esa* its levels did not rise until T11 (Fig. S8). This is consistent with our macroscopic observations and indicates that also on a molecular level *Aly* responds to drought stress most sensitively. Importantly, the observation that all species show transcriptional adjustments to water deficit at T5 indicates that all have perceived the altered water availability, and consequently that the observed response differences are encoded in the downstream signaling network.

Only one gene encoding the cell wall‐localized lipid transfer protein 4 (LTP4; AT5G59310) was commonly induced at T5 in all three species (Fig. 4b; Table S2). This gene was previously shown to be strongly induced by ABA (Gao *et al*., 2016). In *Ath* LTP4 interacts with RACK1, a negative regulator of ABA signaling, which was suggested integrates environmental stress with photosynthesis (Guo *et al*., 2009; Kundu *et al*., 2013). Thus, while the precise placement of LTP4 in the ABA network will require additional studies, this protein appears to have a conserved function in the earliest drought stress responses.

For subsequent analyses we conducted one‐to‐one orthology assignments and focused on 15 883 EMOs (Table S3), which recapitulated the trends observed for all genes (Fig. 4a; Table S2). The superficial annotation of non‐EMO genes precluded their analysis. We were surprised by the limited, albeit significant, overlap among the commonly regulated EMOs; for example, at T11 136 EMOs were up‐ and 27 EMOs were downregulated in all three species (*P* < 0.001, emp. *P*‐value, Figs 4b, S9a,b). Functionally, the commonly upregulated EMOs were enriched in stress‐related functions such as ABA signaling (Table S2), expectedly reflecting the common drought stress response.

Given the moderate overlap, we wondered whether the same EMOs were induced by the species at different time points or whether each species responds with a specific transcriptional program (Fig. 4c). This analysis revealed evidence for differential timing and species‐specific responses. The former is exemplified by segment ‘e’, which contains 978 genes whose induction is timed differently between *Aly* and *Ath*. Functionally these genes include transcriptional regulators, and vesicle trafficking‐related processes (Table S5). By contrast, three segments contain EMOs that are regulated in a species‐specific manner. *Ath* has the largest number of specific EMOs (2251, segment ‘f’), which are functionally enriched in RNA processing categories such as ‘RNA modification’ (FDR 10^−24^), and embryogenesis‐related terms (e.g. ‘embryo development ending in seed dormancy’, FDR 10^−4^). *Aly*‐specific EMOs (630, segment ‘i’) are highly enriched in protein phosphorylation and signaling proteins (FDR 10^−05^), whereas *Esa*‐specific EMOs (390, segment ‘j’) are moderately enriched in cell wall‐related proteins and transcriptional regulators (FDR 0.08) (Table S5). Thus, on a molecular level the species do exhibit differential timing of commonly regulated EMOs, while more than half (55%) of all 5908 induced EMOs are regulated in a species‐specific manner.

As most segments in Fig. 4(c) contain few genes we conducted a functional analysis for all differentially regulated genes of each species (Fig. 4d). As differential timing may be a decisive aspect for eventual survival, we conducted the same analysis for T11 regulated EMOs (Fig. S10; Table S6), the first time‐point with substantial transcriptional changes. In the total analysis a strong and specific *Ath* response is apparent (Fig. 4d inner circle), characterized by RNA processing (10 terms), proteostasis (eight terms) as well as flowering and embryogenesis (10 terms). The late timing and functions together suggest that a major feature of the *Ath* drought response is *escape* via emergency flowering to secure the next generation. In *Aly* also, three ‘flowering and embryogenesis’ terms are weakly enriched (FDR 0.002–0.03). However, more prominent features are metabolic reprogramming and tissue remodeling (Fig. 4d), suggesting metabolic and physiological adaptation to the stress. Common to all three species are the functional groups ‘stress response’, ‘transcriptional regulation’ and ‘hormone signaling’ (Fig. 4d).

Stress response categories were enriched in all species both in the total and in the focused T11 analysis. The ‘response to water deprivation’ and ‘response to salt’ terms were most significant, while other enriched terms refer to heat, cold, wounding and osmotic stress responses. Most of these stresses result in reduced water availability and the genes may function less specifically in the respective stress than the annotation suggests. Similarly, several transcriptional regulation terms were significantly enriched among all species, although with different timing. In *Aly* and *Esa*, ‘transcriptional regulation’ was highly enriched at T11 (*P* < 0.006; Fisher's exact test) contrasting with T14 in *Ath*. Lastly, rewiring of the hormone signaling network is common to all species, but here also important differences can be detected. Common to all three species is a strong induction of salicylic acid (SA; GO:0009751) signaling proteins (Fig. 4d; Table S5). Given the canonical involvement of SA in defense, this appears surprising. However, recently the central SA response regulator NPR1 was shown to also function in cold stress responses (Olate *et al*., 2018). Thus, it is possible that the common upregulation of SA signaling proteins reflects a high degree of pleiotropy of the respective pathway or hints at effects of drought on plant immunity. With this exception, the transcriptionally modulated phytohormone signaling pathways differ between species (Figs 4d, S10). Additional transcripts for ABA signaling proteins were upregulated in *Ath* and *Aly*, whereas transcripts for the karrikin (KAR) pathway were upregulated in *Ath* and *Esa*. No term related to ethylene was found in any of the species. However, in *Aly* at T11 the l‐methionine salvage pathway was strongly upregulated (Fig. S10; *P* < 10^−4^; Fisher's exact test), and this recycles 5′‐methylthioadenosine, a by‐product of ethylene biosynthesis (Albers, 2009). Moreover in the T11 analysis and in the *Aly*‐specific ‘i’ segment, ‘intracellular signaling’ (*P* < 10^−6^; Fisher's exact) and numerous terms indicating phosphorylation‐ and ubiquitination‐mediated signal transduction were found specifically among the T11 *Aly* regulated genes. The significant enrichment of terms in different signaling systems (kinase, hormone and ubiquitination signaling) indicates that a major element of the *Aly* response is a substantial rewiring of the intracellular signal processing network. Importantly, the observed early growth reduction and reduced cell division phenes of *Aly* were mirrored by six terms related to cell cycle, cell division and growth that were enriched among the *Aly* EMOs at T11 but in none of the other species (Fig. S10; Table S6).

*Metabolism and physiology*: at T11 mobilization of alternative energy sources is clearly initiated in *Aly* and *Ath* although the global analysis suggests that this is done more extensively in *Aly*. Specifically notable at T11 was the upregulation of salvage pathways and mobilization of sugar and lipid resources; upregulation of lipid metabolism was also observed in *Ath* (Fig. S10). KEGG pathways of the specifically regulated EMOs confirmed the importance of metabolic rewiring in *Ath,* where amino acid processes, for example ‘lysine degradation’ (*P* < 10^−7^; FDR) and ‘lipid metabolism’, were upregulated (Table S6). Thus, by T11 *Aly* and *Ath* adjust their respective metabolism and activate alternative energy sources. Conversely, for *Esa* metabolic rewiring appears less critical than physiological adjustments. Cell wall biogenesis‐related GO terms (*P* < 0.03; Fisher's exact test) and the KEGG pathway ‘cutin, suberin and wax biosynthesis’ were most significant (*P* < 10^−5^; FDR) among the *Esa*‐regulated EMOs (Table S6) at T11.

These results support our phenotypic observation showing that *Aly* most sensitively responds to lack of water by growth reduction and dramatic intracellular reorganization. By contrast, *Esa* appears prepared even before drought onset and thus requires fewer adjustments. The late *Ath* response is characterized by activation of emergency response mechanisms. Our data further suggest that many response differences are encoded in the signaling network downstream of water deficit perception. Next, we therefore focused on known signaling pathways.

### Regulation of core drought signaling pathways

**Figure 5:**
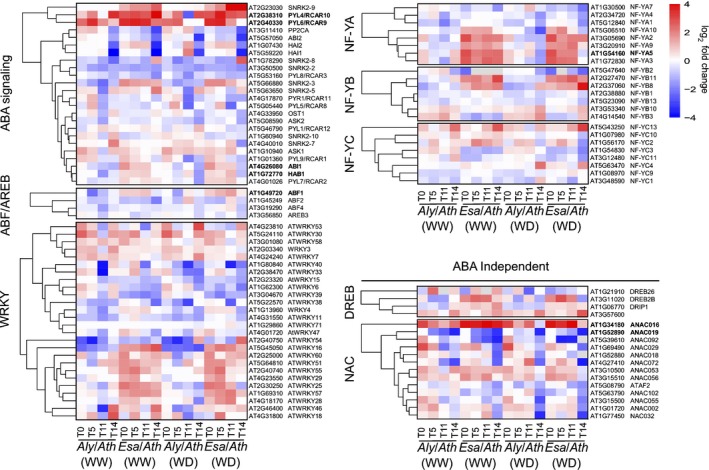
Gene expression dynamics of abscisic acid (ABA) and drought‐stress signaling genes. Heatmaps show relative expression values of genes involved in ABA signaling and selected transcription factors. Color scale represents the fold‐change (log_2_) of *Eutrema salsugineum* (*Esa*) and *Arabidopsis lyrata* (*Aly*) compared to *Arabidopsis thaliana* (*Ath*). Gene annotation is based on *Ath* locus identifiers and annotations (TAIR10). Genes in bold type are discussed in the text.

ABA is the major phytohormone mediating desiccation stress responses (Vishwakarma *et al*., 2017). We started our analysis with ABA signaling proteins in the resistant species relative to *Ath*. In WW conditions several ABA signaling genes were already expressed at higher levels in *Esa* and *Aly*, most notably the orthologs of PYL4/RCAR10 and PYL6/RCAR9 (Fig. 5). Thus, even before the common upregulation in response to drought, several ABA receptors and other signaling proteins show elevated levels in the resistant species. We tested if these expression differences affect stomata function. Consistent with resistant phenotypes and higher expression levels, in normal conditions (no ABA) the stomata of *Aly* and *Esa* plants were less open than those of *Ath* (*R*_WL_ of *c*. 0.40 (*Aly*) and 0.42 (*Esa*) vs *c*. 0.6 for *Ath*). In response to ABA, stomata aperture in *Ath* was reduced by 54% in comparison with mock treatment while in *Aly* and *Esa* the average aperture was reduced by 14% and 12%, respectively (Fig. S11). After ABA stimulation stomata in all species showed similar aperture between 0.32 and 0.37. It is possible that a smaller stomata aperture affects the water use efficiency (WUE) of the resistant *Aly* and *Esa*. Importantly, a recent overexpression screen found that higher levels of the *Aly*‐ and *Esa*‐elevated ABA receptors increase *Ath* WUE (Yang *et al*., 2016; Tischer *et al*., 2017), suggesting a causal contribution to *Aly* and *Esa* drought resistance. While the functional orthology of the *Aly* and *Esa* proteins remains to be shown, this possibly convergent evolution of higher ABA receptor levels in *Aly* and *Esa* is consistent with their resistant phenotype. However, this contrasts with the diverging growth response dynamics in both species, which are thus probably encoded in the signal‐processing network downstream.

We then analyzed the expression of ABA‐dependent transcription factors (TFs) of the ABRF/ABFs, WRKY and the nuclear factor Y (NF‐Y) families (Rushton *et al*., 2012; Zhao *et al*., 2016). In WW grown *Esa*, ABF1 and NF‐YA5 were expressed at elevated levels. Intriguingly, overexpression of NF‐YA5 in *Ath* has been shown to improve its drought resistance (Li *et al*., 2008) (Fig. 5). Expression levels of some ABA‐independent drought response genes such as dehydration‐responsive element binding protein (DREB) and NAC‐domain containing TF family members were elevated independent of stress treatment in both resistant species. Interestingly, the functionally related ANAC016 and ANAC019, both positive regulators of ABA signaling and leaf senescence, showed anticorrelated expression in the resistant and sensitive species.

From these data a picture of the drought signaling system emerges that is differently tuned in the resistant species relative to *Ath*. Intriguingly, several of the genes that are constitutively expressed at higher levels in the resistant species were shown in *Ath* to increase WUE and drought resistance (Tischer *et al*., 2017). This opens the possibility that other signaling genes expressed at higher levels in *Aly* and *Esa* may have similar beneficial effects. In contrast to the divergent drought response phenes, several of the changes in the signaling network are common to *Aly* and *Esa*.

### Dynamics of biochemical changes upon drought

**Figure 6:**
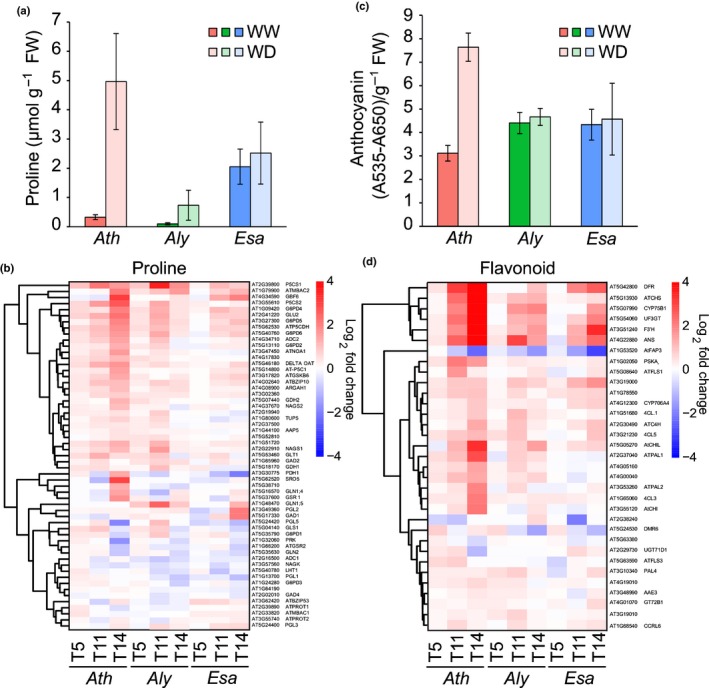
Effect of water stress on proline and anthocyanin accumulation. (a) Proline and (c) anthocyanin content in *Arabidopsis thaliana* (*Ath*), *Arabidopsis lyrata* (*Aly*) and *Eutrema salsugineum* (*Esa*) plants under well‐watered (WW) and water deficit (WD) conditions (T14). Error bars represent SD (*n *=* *4). Heatmap visualization of gene expression levels of proline (b) and flavonoid (d) biosynthesis genes. Color scale represents log_2_ fold change (WD/WW). Gene names are based on *Ath* locus identifiers and annotations (TAIR10).

The phenotypic data suggest an early stress response of *Aly* aimed at reducing water consumption. By contrast, molecular and macroscopic *Esa* responses are less pronounced, suggesting that *Esa* may be in a more drought‐prepared state. Next, we investigated known biochemical drought resistance phenes such as synthesis of the osmoprotectant proline and of the photoprotective scavenger anthocyanin (Hayat *et al*., 2012; Sperdouli & Moustakas, 2012). Slightly elevated basal proline levels that increased in response to salt stress had been reported for *Esa* (Taji *et al*., 2004; Ghars *et al*., 2012). Remarkably, basal proline content in WW‐*Esa* plants was not only several‐fold higher than in *Ath* and *Aly*, but was even nearly three‐fold higher than the stress‐induced levels in *Aly* (Fig. 6a). The biochemical data were partly mirrored by proline metabolic enzyme expression. In all three species expression of P5CS1, encoding a key proline biosynthesis enzyme, peaked at T11 with the strongest regulation observed in *Aly* (Fig. 6b). Consistent with high basal proline levels, *Esa* P5CS1 is expressed at high levels even in unstressed conditions (Taji *et al*., 2004). These data confirm the tempered stress response of *Esa*, and support the interpretation that *Esa* is in a permanent ‘drought ready’ state that requires fewer adjustments upon water scarcity.

Anthocyanin biosynthesis provided a similar picture. Anthocyanin‐metabolism‐related transcripts were upregulated during stress in *Ath* and this upregulation was reflected in a 2.5‐fold increase in anthocyanin levels (Fig. 6c). During drought *Aly* and *Esa* showed a more moderate but clearly discernible upregulation of transcripts; however, the measured anthocyanin levels did not increase by T14 in either species (Fig. 6c,d). Thus, while *Ath* shows signs of oxidative stress, possibly from production of reactive oxygen species (ROS) or increasing intracellular osmolarity, resistant *Aly* and *Esa* have higher basal anthocyanin levels and the transcriptional upregulation of biosynthesis genes does not translate into elevated anthocyanin levels.

Together the molecular data reveal a picture that is more complex than the phenotypic data suggested. While all three species share a common early transcriptional response, subsequent signal processing and response dynamics appear to have diverged, thus giving rise to the contrasting phenotypic manifestations. The data support the conclusion that *Aly* responds more sensitively to lack of water, whereas *Esa* is in a prepared state that requires fewer adjustments in response to drought.

### Clustering analysis reveals a species‐specific mechanism in *Esa*

**Figure 7:**
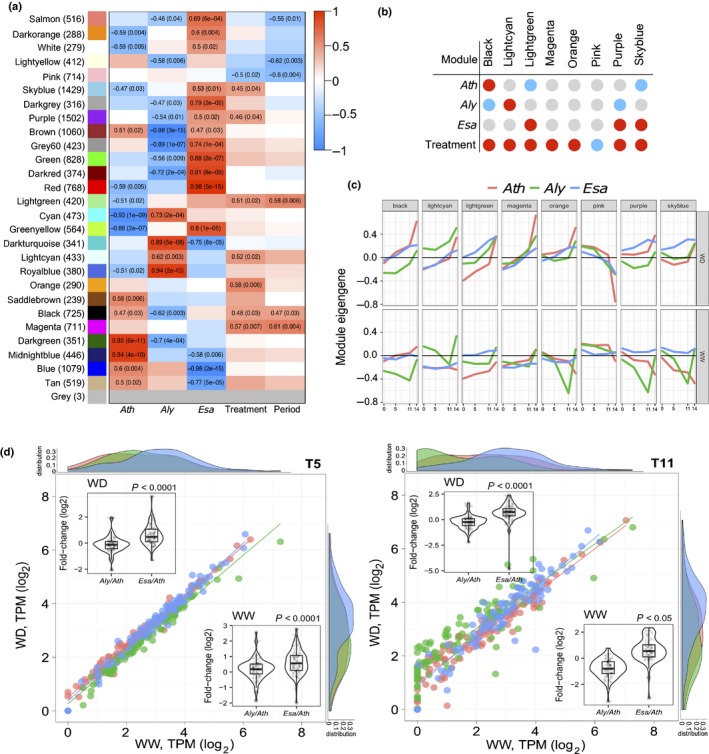
Clustering analysis of expressed mutual orthologs (EMOs). (a) Heatmap showing Pearson correlation between module eigengenes (MEs) and *Arabidopsis thaliana* (*Ath*)*, Arabidopsis lyrata* (*Aly*) and *Eutrema salsugineum* (*Esa*), drought treatment and differential development stages by a weighted gene co‐expression network analysis (WGCNA). Each row corresponds to a module. The number of genes in each module is indicated on the left. Each column corresponds to a trait. Cells show the correlation coefficient (left) and corresponding *P*‐value if significant (right). A threshold parameter of FDR < 0.1 was considered significant. (b) Correlations of significant modules with species and treatment shown as an intersection chart. Red circles indicate positive correlations and blue circles indicate negative correlations. (c) MEs, the first principal component, are calculated to summarize the major vector of gene expression within each module in individual species. Modules with significant association to treatment are shown. (d) Differential histone modification‐associated gene enrichment of *Ath*,* Aly* and *Esa* at T5 and T11 under well‐watered (WW) and water deficit (WD) conditions. Scatter plots show the log_2_TPM values of these genes, density plots show the distribution of log_2_TPM values, and violin plots show the log_2_ fold‐change.

After the targeted analyses we aimed for an unbiased systems approach to analyse the molecular drought responses using a WGCNA (Langfelder & Horvath, 2008). After merging gene sets with highly correlated Eigengenes (Pearson's correlation coefficient > 0.9), 28 network modules were defined and color labeled. The expression patterns of eight modules were significantly (FDR < 0.05; Benjamini–Hochberg correction (BH)) correlated to drought treatment and thus likely represent different features of the stress response (Fig. 7a–c). Of these eight modules only one was correlated with both resistant species (Fig. 7b), but negatively correlated with *Aly* and positively with *Esa*.

We queried the biological significance of these modules by exploring gene function (GO) and pathway (KEGG) enrichment (Figs S12a–h, S13a–h; Table S7). The module negatively associated with treatment (pink) was strongly enriched in terms describing photosynthetic processes (Figs S12f, S13f), thus corresponding to the downregulation of photosynthetic processes. Species‐independent and positively associated with drought were the magenta and orange modules (Fig. 7b). Genes in the magenta module were enriched in drought response functions such as ‘water transport’, ‘stomatal movement’ and ‘anthocyanin metabolism’ (Fig. S12d). KEGG pathway analysis additionally revealed altered mitogen‐activated protein kinase signaling increased catabolism of fatty acids and amino acids, and redirection of vesicle traffic (Fig. S13d). Many of these changes, which are most pronounced in *Ath* and least in *Esa*, probably serve to activate energy reserves to compensate for the reduction of photosynthetic activity. The orange module is dominated by nucleic acid, DNA and protein‐related metabolic and transport processes, whereas significant KEGG pathways included several lipid catabolism pathways (Figs S12e, S13e).

We then focused on modules associated with individual species to define specific responses. The module positively correlated with *Aly* (lightcyan) contained mostly poorly or unannotated EMOs such that no meaningful analysis was possible (Figs S12b, S13b). Positively correlated with *Esa* were the purple and sky‐blue modules. The purple module contained genes in several recycling‐related categories including autophagy (*P* < 10^−4^; Fisher's exact) and vacuole organization indicating that *Esa* also had to cope with energy deprivation. Striking in both modules was the enrichment of mRNA processing functions, for example spliceosome (*P* < 10^−4^, Fisher's exact test) and mRNA surveillance (FDR < 10^−5^, BH), suggesting that alternative splicing may play an important role in *Esa* drought response. Fascinatingly, while the Eigengenes for the EMOs in these modules indicate their expression in *Esa* throughout development and only a moderate upregulation in response to drought, genes with similar functions are strongly upregulated in *Ath* at T14. Also remarkable was the enrichment of all major DNA repair pathways, that is ‘nonhomologous end‐joining’, ‘nucleotide excision repair’ and ‘homologous recombination’ (all FDR < 0.05, BH) (Figs S12g,h, S13g,h), and the GO term ‘DNA repair’ (*P* < 10^−4^; Fisher's exact test). We wondered whether this was a consequence of an increased production of ROS. However, at T14 we saw a dramatic decline of H_2_O_2_ levels in WD *Esa* plants compared to WW controls (not shown), making stress‐induced ROS‐mediated DNA damage less likely. In animals the DNA damage response is closely linked to chromatin remodeling (Hauer & Gasser, 2017). In fact, we found several terms related to epigenetic reprogramming and DNA organization enriched in the sky‐blue module, which is positively correlated with drought treatment and *Esa*, for example ‘chromatin remodeling’, ‘chromatin organization’ and ‘histone acetylation’ (all *P* < 10^−4^; Fisher's exact test). Moreover, at T5 and T11 histone‐modifying genes were expressed at substantially higher levels in *Esa* than in *Aly* and in *Ath,* providing additional support for an important role of epigenetic programming in *Esa* drought stress resistance (Fig. 7d; Table S8).

## Discussion

Drought resistance is a complex phenotype shaped by the interplay of varied physiological and underlying molecular processes. The diversity of involved response phenes poses a challenge for our understanding of drought resistance. We aimed to understand the physiological and molecular changes that contribute to increased drought resistance within Brassicaceae using *Ath*,* Aly* and *Esa* as representative models.

As water requirements depend strongly on the developmental stage, we first synchronized developmental timelines. Leaf formation was most different between the species up to stage 1.03 and progressed much more synchronously afterwards. By starting drought treatment at 1.06 we were thus able to reduce the impact of developmental effects on the measured drought phenotypes. Intriguingly, this carefully controlled experimental set‐up revealed similar drought resistance, as measured by *Aly* and *Esa* survival; at the same time, the observed level of resistance was less striking than expected from previous reports. Both of these findings reiterate the importance of a carefully controlled experimental set‐up.

Our subsequent phenotypic analysis suggested different response strategies of the two resistant species. Several growth‐related parameters indicate that *Aly* reduces leaf growth even by 72 h after treatment predominantly via reduction of cell proliferation, whereas *Esa* primarily reduces cell growth. However. these adjustments are detectable only 144 h after treatment and are thus even more delayed than the drought‐sensitive *Ath*. As soil–water content decreased identically across the drought treatment in pots of all three species, it can be excluded that the response differences are due to differences in water consumption. These data suggest that *Aly* and *Esa* utilize different strategies to achieve the same level of drought resistance.

To gather support for this preliminary conclusion and more detailed insight into the molecular response mechanisms we conducted detailed transcriptional profiling of all three species. Similar to observations for growth phenes, *Aly* exhibited the earliest strong transcriptional response at T11, followed by *Ath* at T14. Compared to these two, *Esa* transcriptional responses were more moderate, but did peak also at T11. Similarly, in *Aly* the drought stress marker RD29B was already upregulated at T5, whereas in *Ath* and *Esa* it was first detectable at T11. These data further support the conclusion that *Aly* triggers molecular and phenotypic stress response mechanisms much earlier than both *Ath* and *Esa*. Two mutually not exclusive explanations could account for this phenomenon: either *Ath* and *Esa* sense the water deficit later than *Aly* but respond with similar kinetics once they do, or the three species perceive the water deficit with similar sensitivity but their signaling and response networks are tuned to trigger the stress responses more rapidly or delayed, respectively. Naturally, the answer to this question affects which kind of biotechnological adaptations would most effectively increase the tolerance of a sensitive species. The transcriptional changes of similar magnitude at T5 and especially the upregulation of the ABA‐responsive LTP4 at T5 in all three species indicate that water deficit perception is similarly sensitive in all three species. Consequently, this implies that the response differences are at least partially encoded in the downstream signal processing and response machinery.

In this context, it is noteworthy that despite their contrasting response patterns even in unstressed conditions several ABA receptors, PP2Cs and TFs are expressed at higher levels in *Aly* and *Esa* relative to *Ath*. Intriguingly, several of the respective *Ath* orthologs were recently shown to increase WUE and drought resistance when overexpressed in *Ath*. This could suggest that elevated expression of other genes upregulated in the resistant species may have similar effects. As a caveat, even though the phylogenetic analysis clearly identifies the involved genes as orthologs, experimental validation of the functional orthology as well as validation of the beneficial effects of additional genes will be important next steps. It is interesting that one of the *Esa‐* and *Aly*‐upregulated proteins, PYL6, was the only remaining ABA receptor in a duodecuple mutant, and is able to partially activate ABA transcriptional responses (Zhao *et al*., 2018).

In addition to these common changes, a dramatic drought‐induced rewiring of the signal transduction network was observed in all species by T11. At this stage it is unclear what proportion of these changes are part of the acute drought stress response and to what extent the adjustments, for example of signaling pathways, relate to naturally occurring environmental conditions, namely repeated drought periods or persistent low water availability. Also shared between the resistant species is the massive transcriptional reprogramming at T11, when nearly 15% of *Aly* and *Esa* differentially regulated EMOs function in ‘transcriptional regulation’. Given the overall moderate transcriptional changes in *Esa*, however, it is possible that several of these transcriptional changes may mediate escape or adaptation mechanisms in other environmental scenarios than the one tested here. Overall these analyses suggested that all three species perceived lack of water similarly early, but in each species the downstream signal processing networks are wired differently, thus giving rise to the specific responses. Consequently, a more detailed understanding of basal and stress‐triggered signal processing networks will be required to understand which specific network features underlie the different response strategies.

A common stress response was downregulation of photosynthesis and activation of alternative energy sources, which are required because stomatal closure, which reduces evaporative water loss, also prevents uptake of CO_2_. Other well‐described adaptations to water deprivation are synthesis of proline and flavonoids as osmoprotectants and scavengers. The relatively late but strong responses suggest that *Ath* may respond too slowly and then quickly enters an emergency mode. By contrast, *Aly* responds most sensitively to drought by adjusting growth, metabolism, signaling and transcriptional programs. *Esa* appears to perceive decreasing water availability as sensitively as the other two species. Possibly due to permanent ‘preparatory adjustments’, fewer adjustments such as cell wall remodeling are necessary compared to the other species. More intriguing was the upregulation of splicing, DNA repair and epigenetic programming transcripts in *Esa*, the specific role of which remains to be elucidated.

In conclusion, our results showed that phenotypic and morphological changes of plants under drought stress can be subtle, although well‐controlled and detailed studies may identify important differences that will be important for a systems‐level understanding of drought stress resistance. Conceptually, to understand individual phenes and underlying molecular mechanisms a deep phenotyping of plants in different environmental conditions is required. Our study indicates that a key difference between Brassicaceae is most likely encoded in the signal transduction network downstream of initial water deficit perception. Thus, future studies will need to focus on charting the molecular network connectivity and model dynamics of the drought stress signal transduction network.

## Author contributions

PF‐B conceived the project; NMdlR, SD, NG, DI and PF‐B designed experiments; NMdlR, SD and NG performed experiments and analyzed the data; C‐WL, JYK and PF‐B conceived and conducted bioinformatics analysis and statistics; NMdlR, C‐WL and PF‐B wrote the manuscript. NMdlR and C‐WL contributed equally to this work.

## Supporting information

Please note: Wiley Blackwell are not responsible for the content or functionality of any Supporting Information supplied by the authors. Any queries (other than missing material) should be directed to the New Phytologist Central Office.

**Fig. S1** Effects of severe water deficit on photosynthetic efficiency and survival.
**Fig. S2** Growth rate dynamics.
**Fig. S3** Rosette morphology parameters.
**Fig. S4** Reduction in area of detached leaves at T11.
**Fig. S5** Measurements of stomatal parameters.
**Fig. S6** Dynamics of transcriptional changes.
**Fig. S7** Dynamics of transcriptional changes and their GO‐based functional classification.
**Fig. S8** Drought stress response gene expression.
**Fig. S9** Significant overlap of differentially regulated genes.
**Fig. S10** GO terms enriched at T11.
**Fig. S11** Stomatal aperture in response to ABA.
**Fig. S12** GO‐based functional classification of treatment‐associated modules.
**Fig. S13** KEGG‐based functional classification of treatment‐associated modules.

**Table S1** Overview of phenotypic characteristics.

**Table S2** Differential regulated genes and GO enrichment.

**Table S3** Ortholog relationships and complete expression matrix.

**Table S4** Dynamics of transcriptional changes of drought‐induced genes.

**Table S5** Significant GO terms of dynamic transcriptional changes.

**Table S6** Not shared GO terms and KEGG pathways at T11.

**Table S7** Significant GO terms and KEGG pathways of modules.

**Table S8** Gene abundance of histone modification genes.
