# Supplementary material for: Drought resistance is mediated by divergent strategies in closely related Brassicaceae
Source: New Phytol. 2019 Jun 18;223(2):783–97. doi: 10.1111/nph.15841 (PMC6771540; doi:10.1111/nph.15841)
Supplement: Supplementary file 1 — Fig. S1 Effects of severe water deficit on photosynthetic efficiency and survival. Fig. S2 Growth rate dynamics. Fig. S3 Rosette morphology parameters. Fig. S4 Reduction in area of detached leaves at T11. Fig. S5 Measurements of stomatal parameters. Fig. S6 Dynamics of transcriptional changes. Fig. S7 Dynamics of transcriptional changes and their GO‐based functional classification. Fig. S8 Drought stress response gene expression. Fig. S9 Significant overlap of differentially regulated genes. Fig. S10 GO terms enriched at T11. Fig. S11 Stomatal aperture in response to ABA. Fig. S12 GO‐based functional classification of treatment‐associated modules. Fig. S13 KEGG‐based functional classification of treatment‐associated modules. [file NPH-223-783-s001.pdf]

***New Phytologist* Supporting Information**

Article title: **Drought resistance is mediated by divergent strategies in closely related Brassicaceae**

Authors: Nora Marín-de la Rosa, Chung-Wen Lin, Yang Jae Kang, Stijn Dhondt, Nathalie Gonzalez, Dirk Inzé and Pascal Falter-Braun.

Article acceptance date: 29 March 2019

Supporting Information

Fig. S1 Effects of severe water deficit on photosynthetic efficiency and survival.
Fig. S2 Growth rates dynamics.
Fig. S3 Rosette morphology parameters.
Fig. S4 Reduction in area of detached leaves at T11.
Fig. S5 Measurements of stomatal parameters.
Fig. S6 Dynamic of transcriptional changes.
Fig. S7 Dynamic of transcriptional changes and their GO-based functional classification.
Fig. S8 Drought stress response gene expression.
Fig. S9 Significant overlap of differentially regulated genes.
Fig. S10 GO terms enriched at T11.
Fig. S11 Stomatal aperture in response to ABA.
Fig. S12 GO-based functional classification of treatment associated modules.
Fig. S13 KEGG-based functional classification of treatment associated modules.

Table S1 Overview of phenotypic characteristics (separate file).
Table S2 Differential regulated genes and GO enrichment (separate file).
Table S3 Orthologue relationships and complete expression matrix (separate file).
Table S4 Dynamic of transcriptional changes of drought-induced genes (separate file).
Table S5 Significant GO terms of dynamic transcriptional changes (separate file).
Table S6 Not shared GO terms and KEGG pathways at T11 (separate file).
Table S7 Significant GO terms and KEGG pathways of modules (separate file).
Table S8 Gene abundance of histone modification genes (separate file).

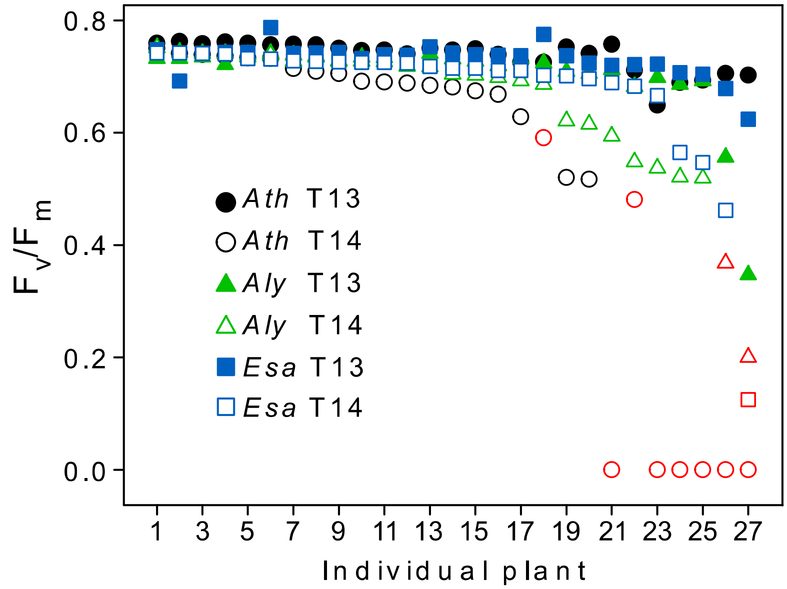

Fig. S1 Effects of severe water deficit on photosynthetic efficiency and survival. F_v/F_m ratio of individual plants at T13 and T14 (just before re-watering); red labeled individuals did not recover 3 days after re-watering. *Ath, Arabidopsis thaliana*. *Aly, Arabidopsis lyrata. Esa, Eutrema salsugineum.*

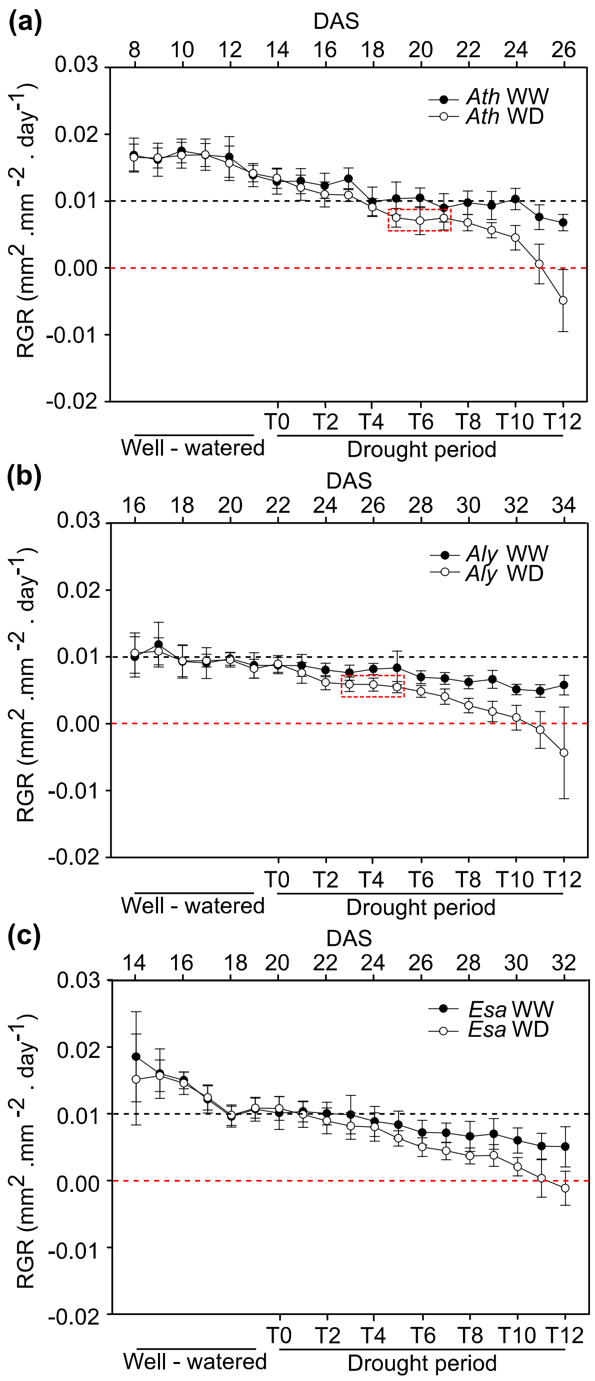

Fig. S2 Growth rates dynamics. Relative growth rate (RGR) in (a) *Arabidopsis thaliana* (*Ath*), (b) *Arabidopsis lyrata* (*Aly*) and (c) *Eutrema salsugineum* (*Esa*). Dots below the red dotted line are considered as negative growth rates. Red dotted box marks the first day of significant growth reduction and the two consecutive days in *Ath* and *Aly*. Data are represented as mean ± SD. DAS, days after sowing. WW, well-watered. WD, water deficit.

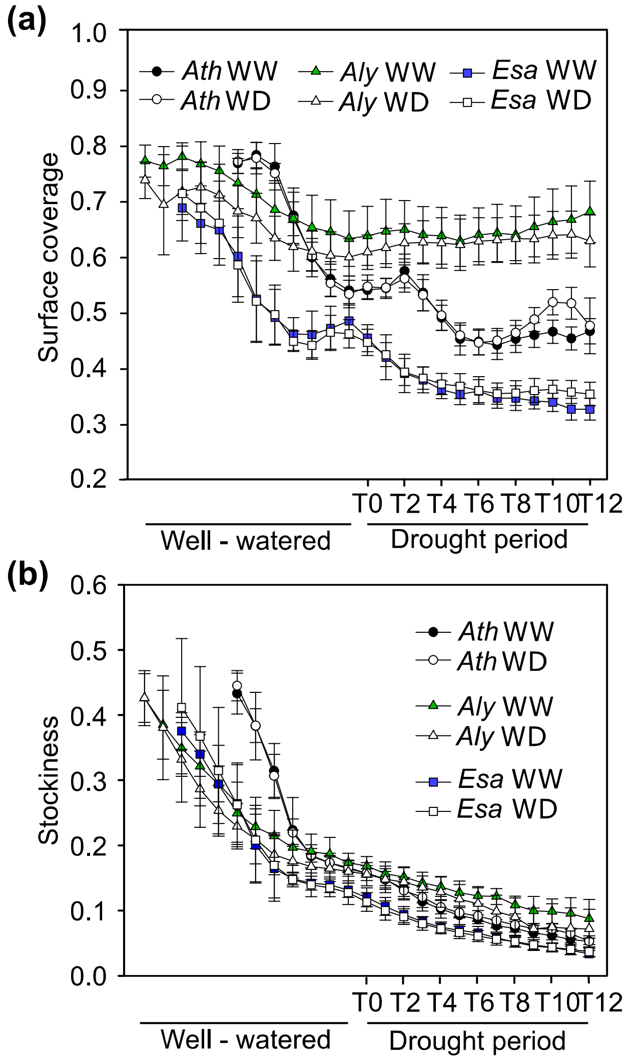

Fig. S3 Rosette morphology parameters. (a) Rosette surface coverage over time. Measure indicates occupancy of the rosette in the convex hull area. (b) Stockiness over time. Measure denotes rosette roundness. $n > 25$ plants per time point and treatment; data are represented as mean ± SD. WW, well-watered. WD, water deficit. *Ath, Arabidopsis thaliana*. *Aly, Arabidopsis lyrata. Esa, Eutrema salsugineum.*

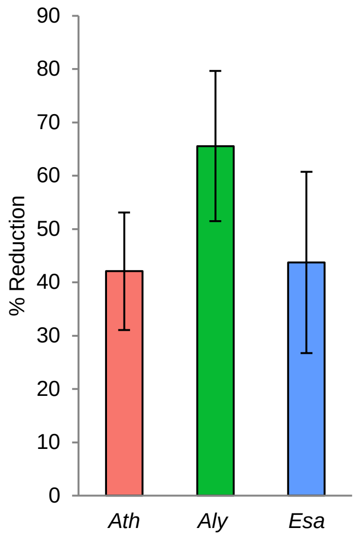

Fig. S4 Reduction in area of detached leaves at T11. The decrease in leaves area is expressed as percent. (*n* = 10 plants per species and treatment; data are represented as mean ± SD). *Ath, Arabidopsis thaliana*. *Aly, Arabidopsis lyrata. Esa, Eutrema salsugineum.*

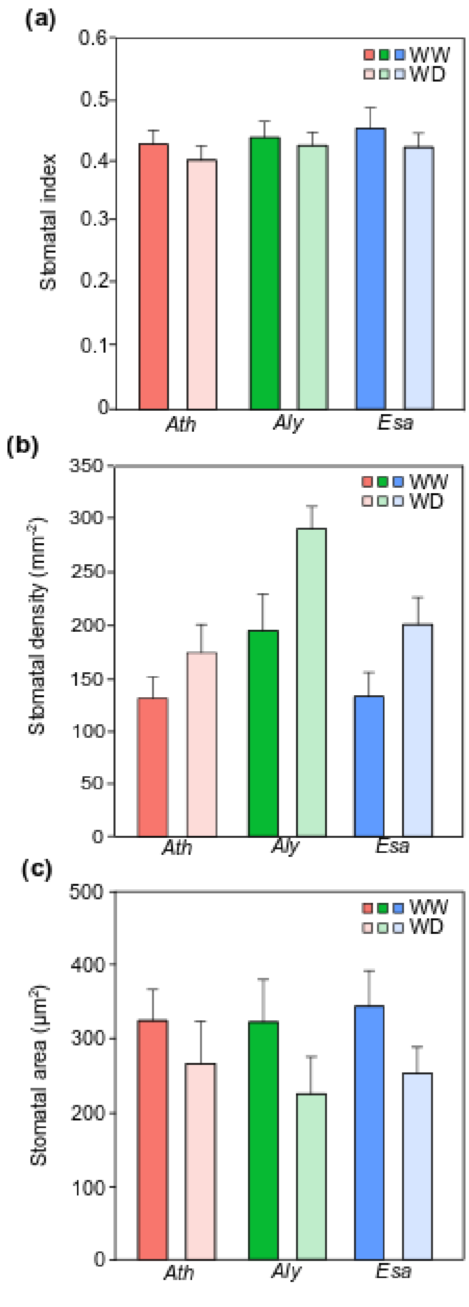

Fig. S5 Measurements of stomatal parameters. (a) Stomatal index, (b) stomatal density and (c) stomatal area of leaf six at WW (well-watered) and WD (water deficit) conditions (n = 5 - 8 plants per species and treatment). Data are represented as mean ± SD. *Ath, Arabidopsis thaliana*. *Aly, Arabidopsis lyrata. Esa, Eutrema salsugineum.* Numeric data provided in Table S1.

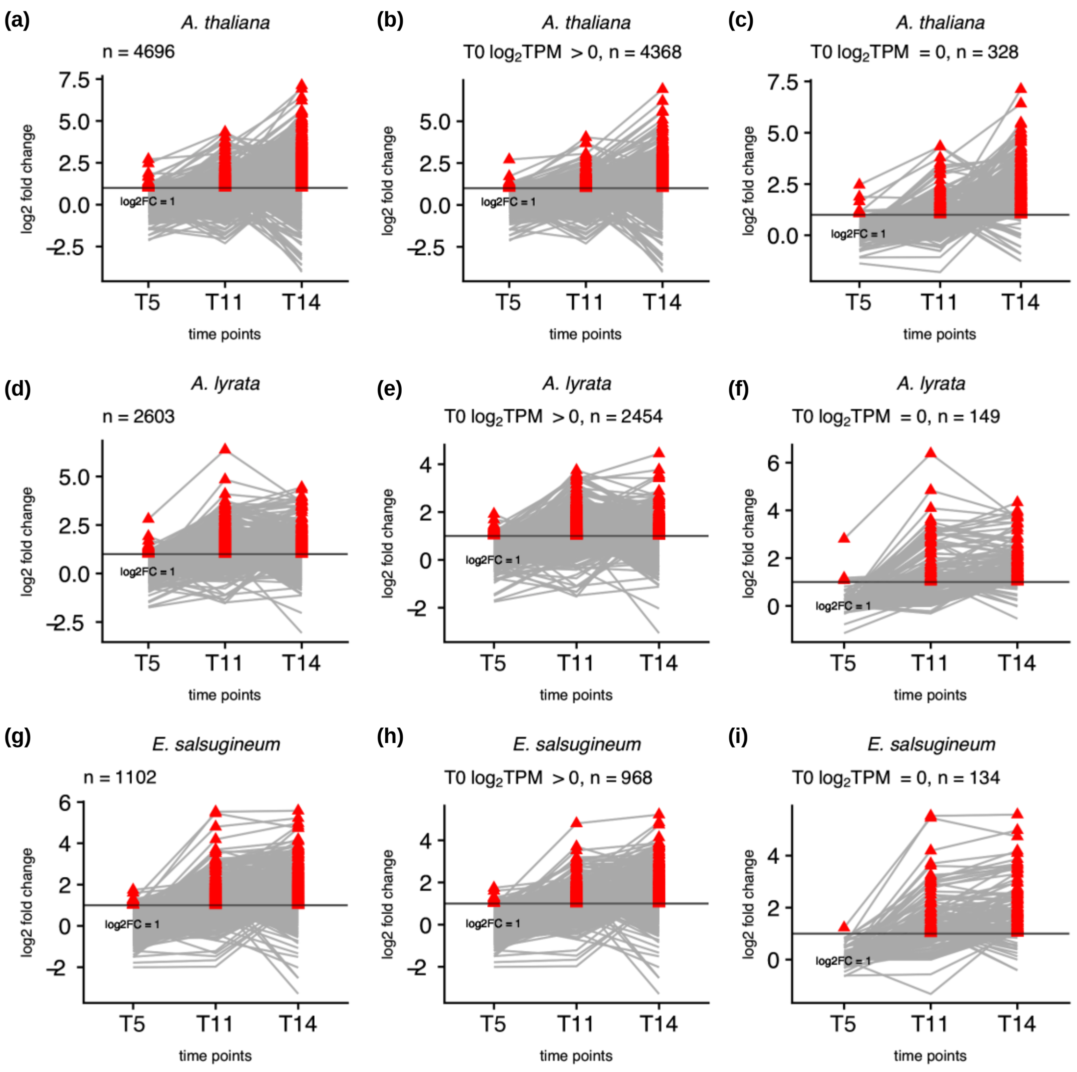

Fig. S6 Dynamic of transcriptional changes. Upregulated genes (T5-T14) were classified into not expressed at T0 (log2TPM = 0; basal -) and expressed at T0 (log2TPM > 0; basal +). *Ath* upregulated (a), *Ath* basal + (b), *Ath* basal - (c), *Aly* upregulated (d), *Aly* basal + (e), *Aly* basal - (f), *Esa* upregulated (g), *Aly* basal + (h), *Aly* basal - (i).

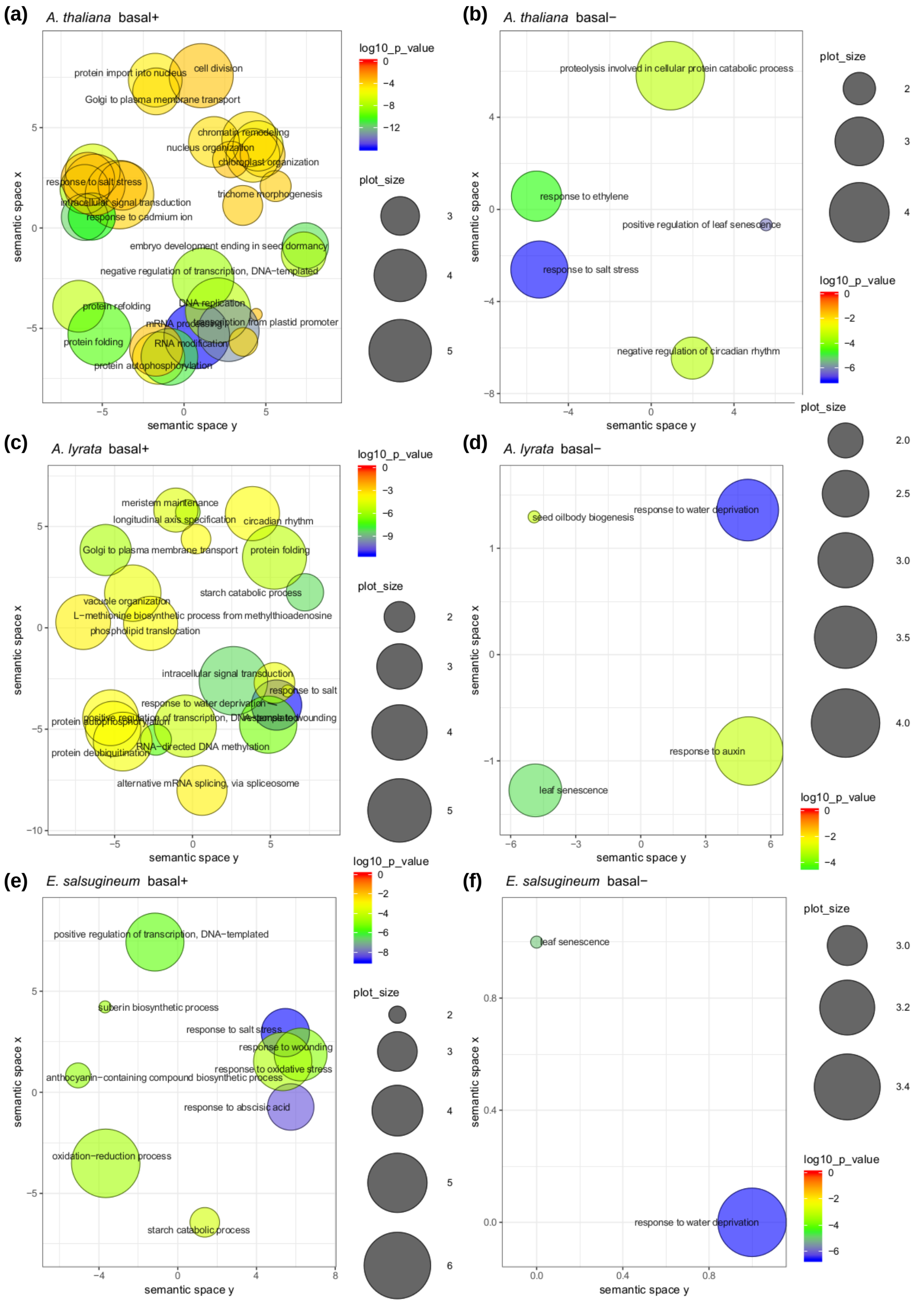

Fig. S7 Dynamic of transcriptional changes and their GO-based functional classification. Gene ontology classifications visualized as scatter plot. Summarized GO terms related to biological processes at baseline. Circle color indicates the log10 *p*-value and circle size indicates the frequency of the GO term in the underlying EBI GOA databases. The scatter plots represent *Ath* basal + (a), *Ath* basal - (b), *Aly* basal + (c), *Aly* basal - (d), *Esa* basal + (e), *Esa* basal - (f).

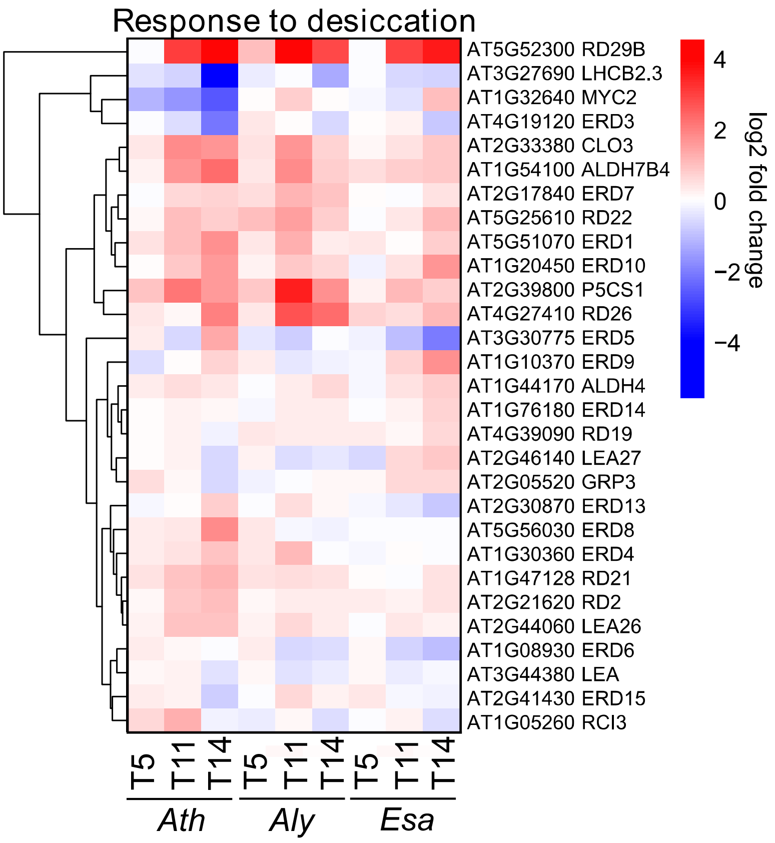

Fig. S8 Drought stress response gene expression. Heatmap visualization of expression levels for "response to desiccation" (RD) and "early-response to desiccation" (ERD) gene families, and genes annotated with GO term "response to desiccation" (GO:0009269). Color scale represents log2 fold change (WD/WW). Gene names are based on *Arabidopsis thaliana* (*Ath*) locus identifiers and annotation (TAIR10). WW, well-watered. WD, water deficit. *Aly, Arabidopsis lyrata. Esa, Eutrema salsugineum.*

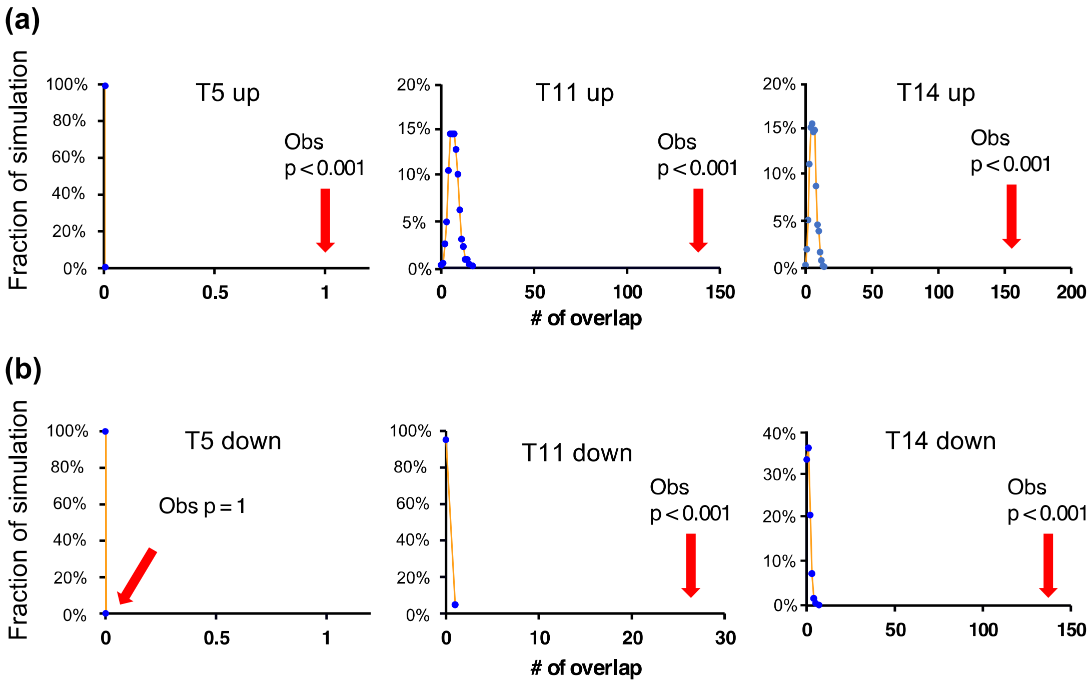

Fig. S9 Significant overlap of differentially regulated genes. Random permutation simulation showing the significance of observed overlap between *Arabidopsis thaliana* (*Ath*), *Arabidopsis lyrata (Aly*) and *Eutrema salsugineum* (*Esa)* (a) up- and (b) down-regulated genes at T5, T11 and T14.

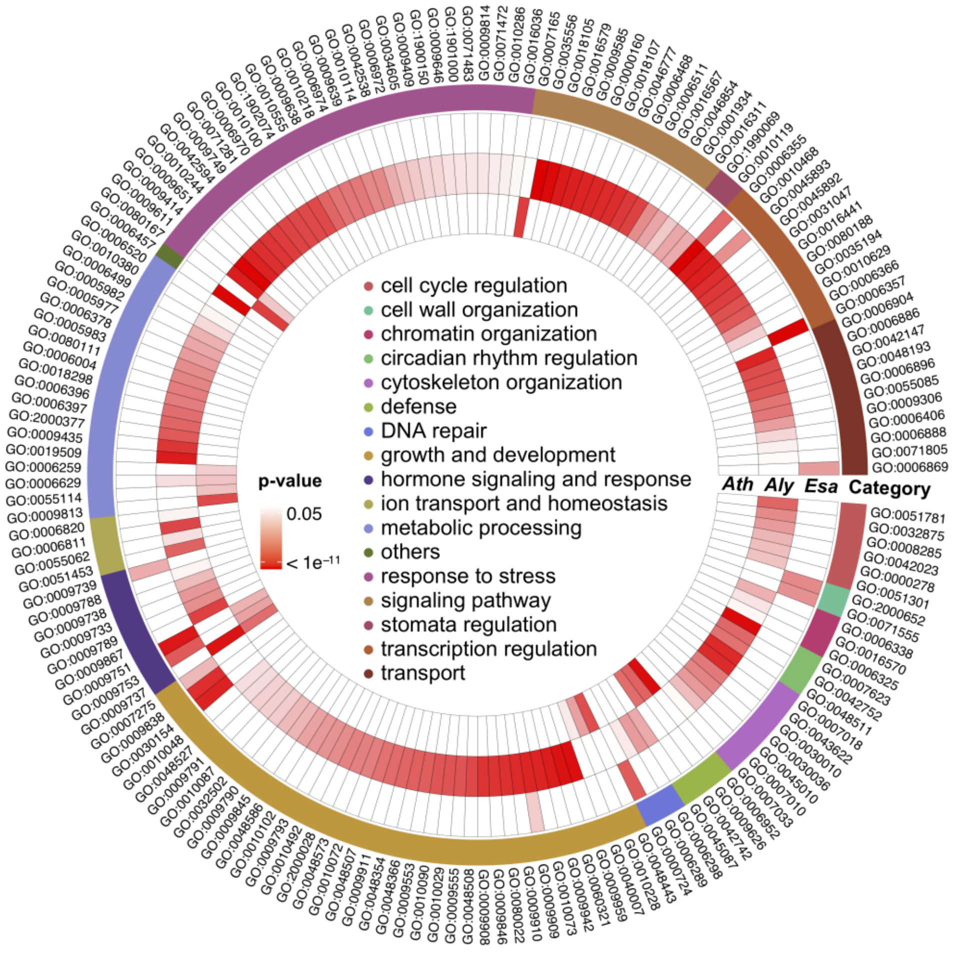

Fig. S10 GO terms enriched at T11. GO terms enriched in the three EMO sets specifically regulated in each species at T11 (p-value; Fischer´s exact). *Ath*, *Arabidopsis thaliana*. *Aly, Arabidopsis lyrata. Esa, Eutrema salsugineum.* Numeric Data in Table S6.

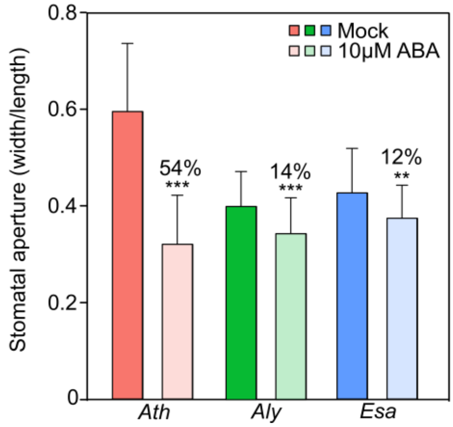

Fig. S11 Stomatal aperture in response to 10 µM ABA. “%” indicates relative decrease of ABA treated plants relative to mock. Data are represented as mean ± SD (***P*<0.01; ****P*<0.001, Student’s t-test). *n* > 40 stomata per treatment. *Ath*, *Arabidopsis thaliana*. *Aly, Arabidopsis lyrata. Esa, Eutrema salsugineum.*

Fig. S12 GO-based functional classification of treatment associated modules. Gene ontology classifications of significant modules visualized as scatter plot. Summarized GO terms related to biological processes at baseline. Circle color indicates the log10 p-value and circle size indicates the frequency of the GO term in the underlying EBI GOA databases. The scatter plots represent black (a), lightcyan (b), lightgreen (c), magenta (d), orange (e), pink (f), purple (g) and skyblue (h) module.

(a)

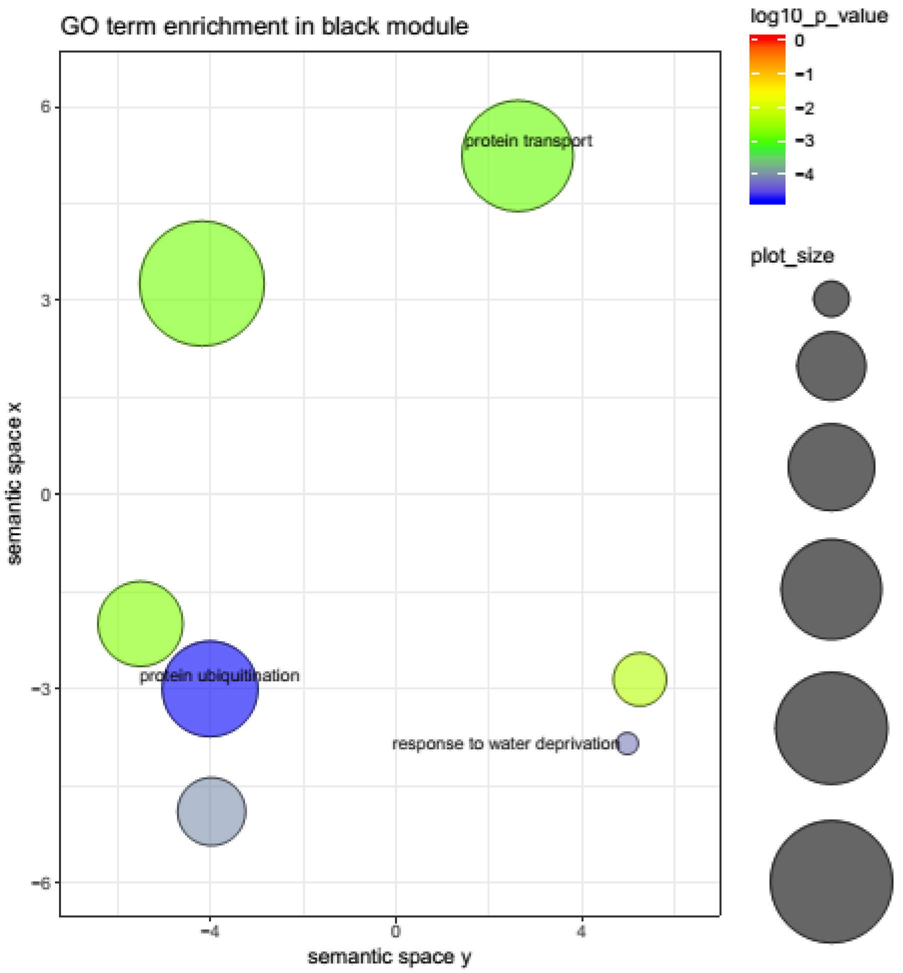

(b)

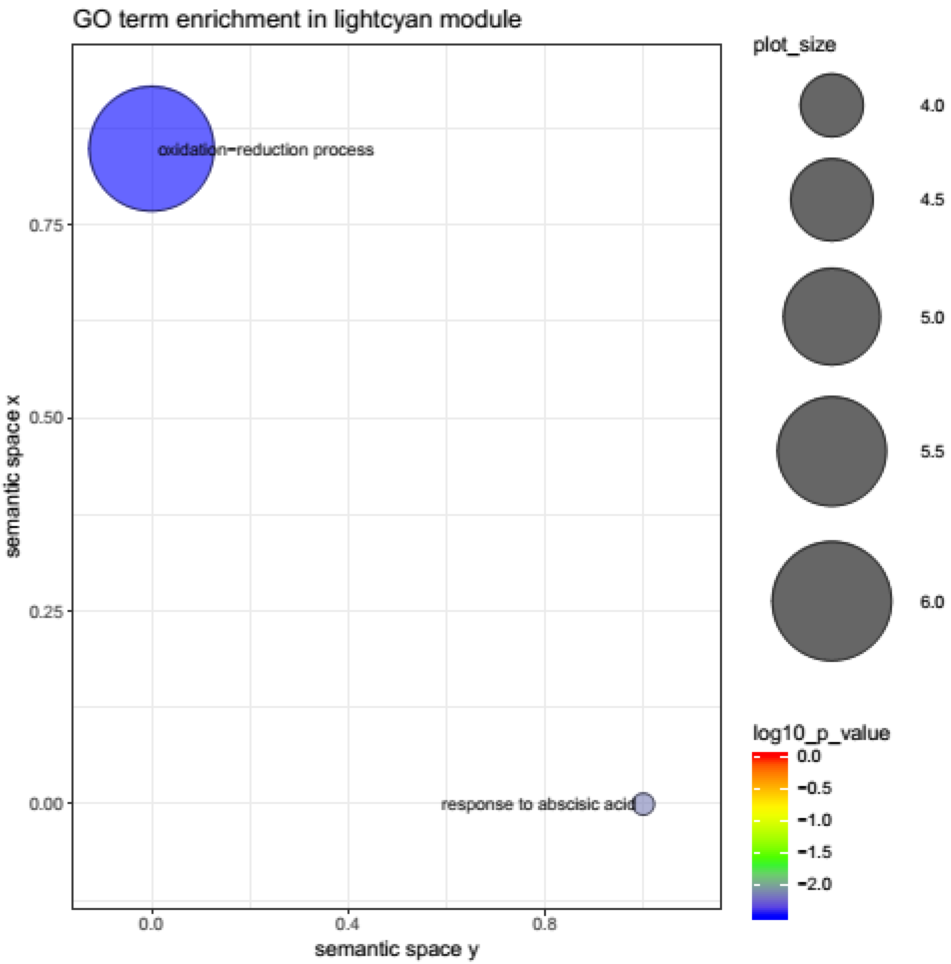
GO term enrichment in lightcyan module
oxidation−reduction process
response to abscisic acid
semantic space x
0.75
0.50
0.25
0.00
0.0
0.4
0.8
semantic space y
plot_size
4.0
4.5
5.0
5.5
6.0
log10_p_value
0.0
−0.5
−1.0
−1.5
−2.0

(c)

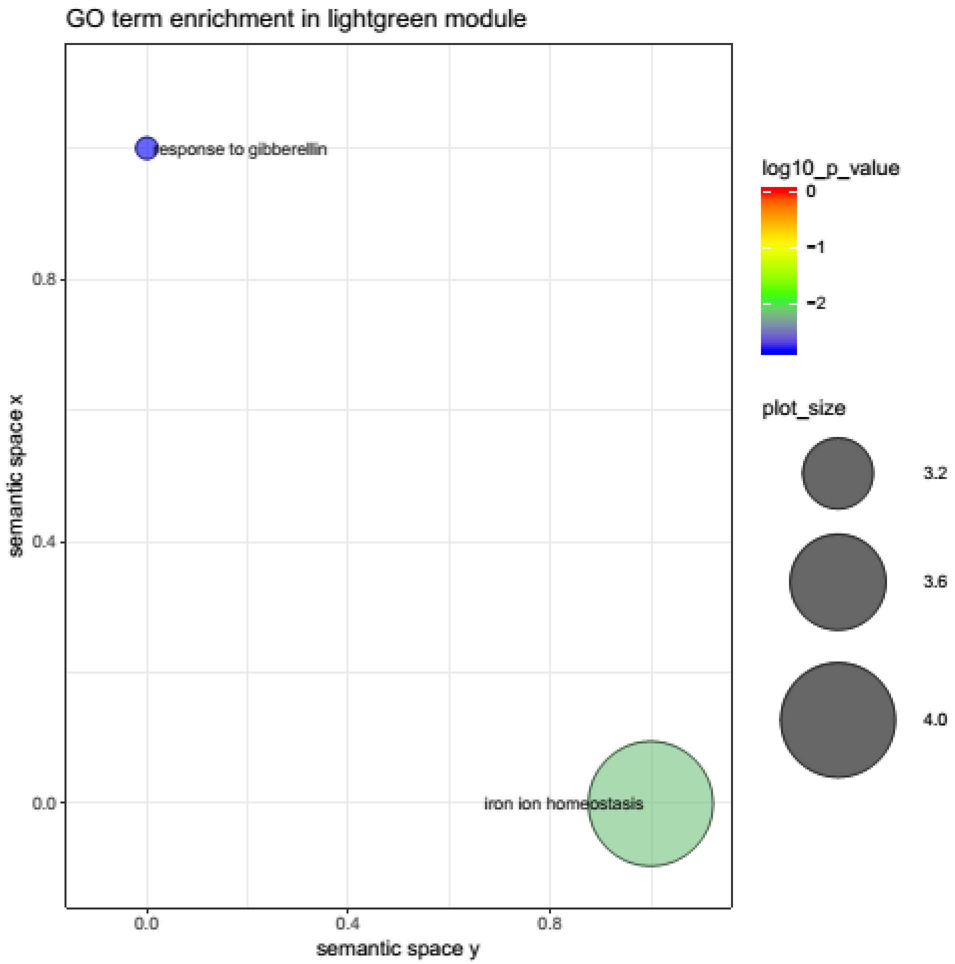

GO term enrichment in lightgreen module
response to gibberellin
iron ion homeostasis
0.8
0.4
0.0
semantic space x
0.0
0.4
0.8
semantic space y
log10_p_value
0
-1
-2
plot_size
3.2
3.6
4.0

(d)

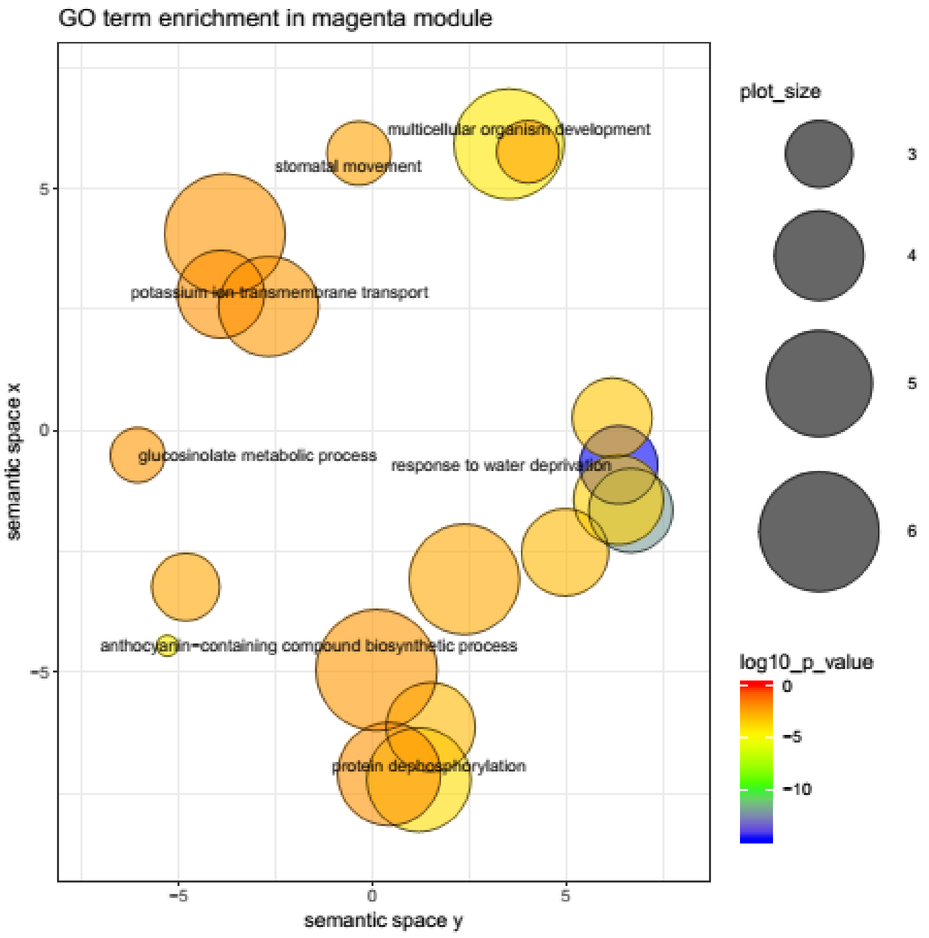
GO term enrichment in magenta module
multicellular organism development
stomatal movement
potassium ion transmembrane transport
glucosinolate metabolic process
response to water deprivation
anthocyanin−containing compound biosynthetic process
protein dephosphorylation
semantic space x
semantic space y
5
0
−5
plot_size
3
4
5
6
log10_p_value
0
−5
−10

(e)

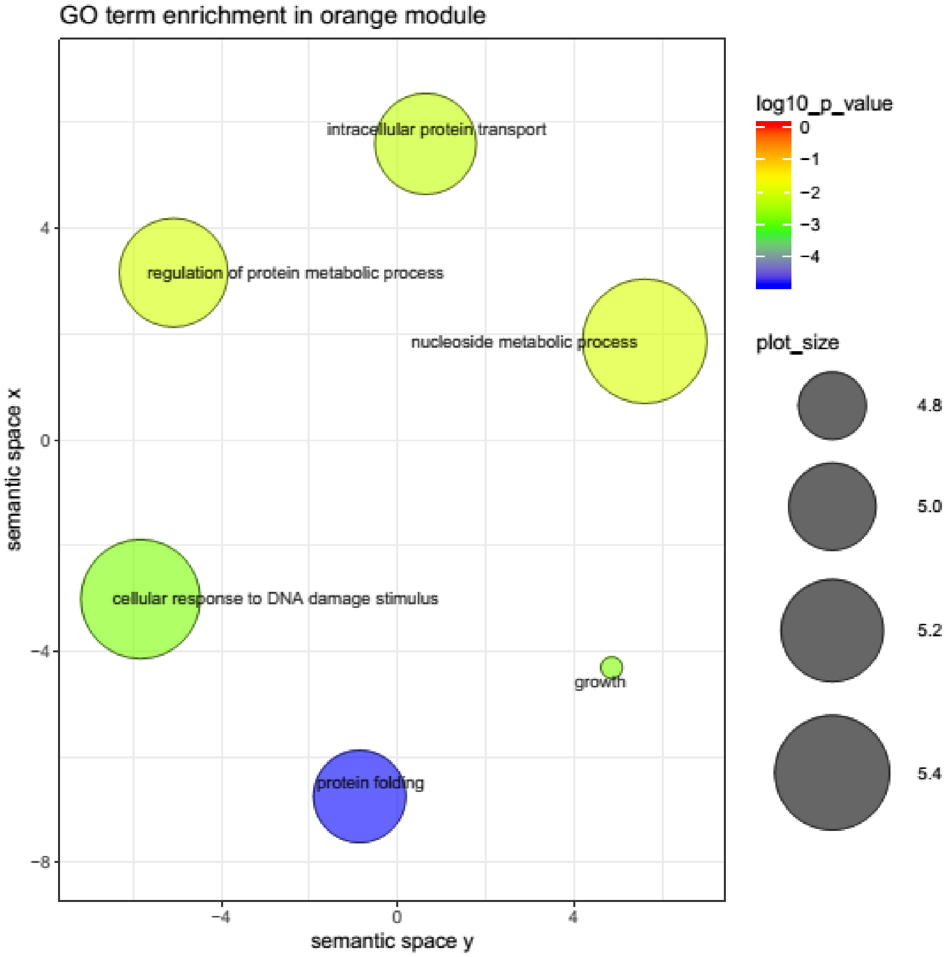

GO term enrichment in orange module
intracellular protein transport
regulation of protein metabolic process
nucleoside metabolic process
cellular response to DNA damage stimulus
growth
protein folding
semantic space x
semantic space y
4
0
−4
−8
−4
0
4
log10_p_value
0
−1
−2
−3
−4
plot_size
4.8
5.0
5.2
5.4

(f)

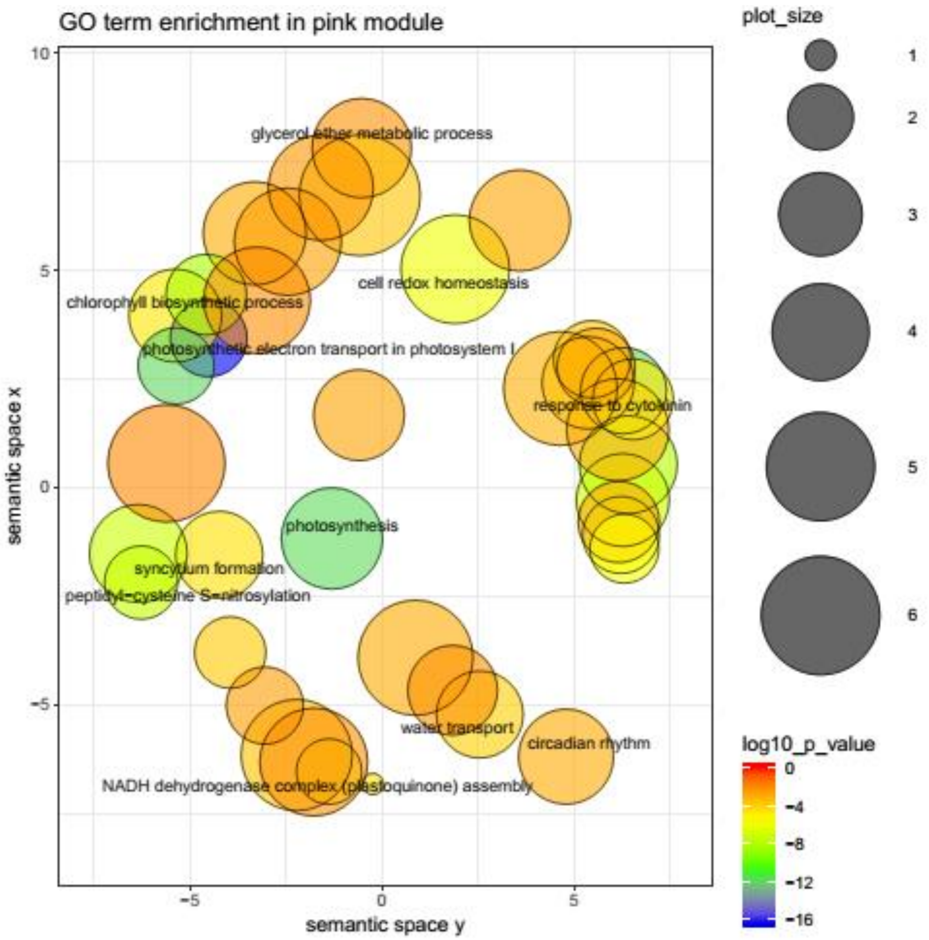

GO term enrichment in pink module
glycerol ether metabolic process
cell redox homeostasis
chlorophyll biosynthetic process
photosynthetic electron transport in photosystem I
response to cytokinin
photosynthesis
syncytium formation
peptidyl-cysteine S-nitrosylation
water transport
circadian rhythm
NADH dehydrogenase complex (plastoquinone) assembly
semantic space x
semantic space y
10
5
0
-5
-5
0
5
plot_size
1
2
3
4
5
6
log10_p_value
0
-4
-8
-12
-16

(g)

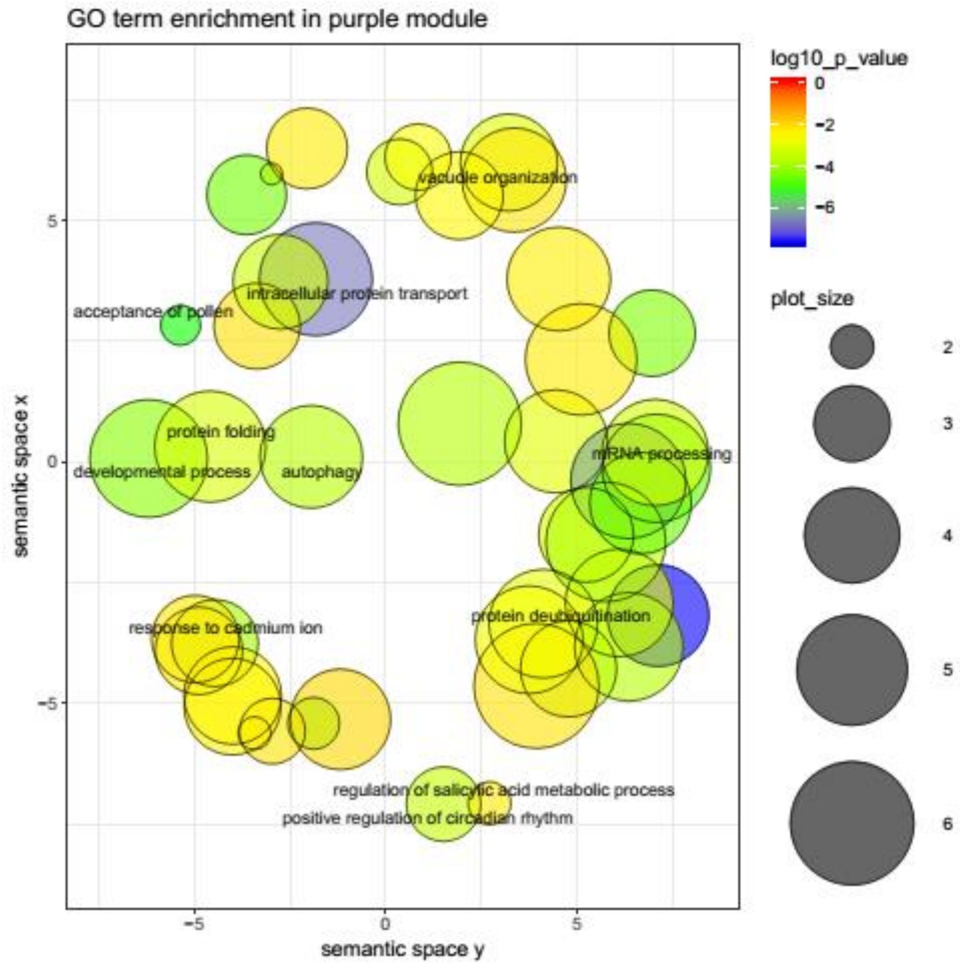
GO term enrichment in purple module
vacuole organization
intracellular protein transport
acceptance of pollen
protein folding
developmental process
autophagy
mRNA processing
protein deubiquitination
response to cadmium ion
regulation of salicylic acid metabolic process
positive regulation of circadian rhythm
semantic space x
semantic space y
5
0
-5
log10_p_value
-2
-4
-6
plot_size
2
3
4
5
6

(h)

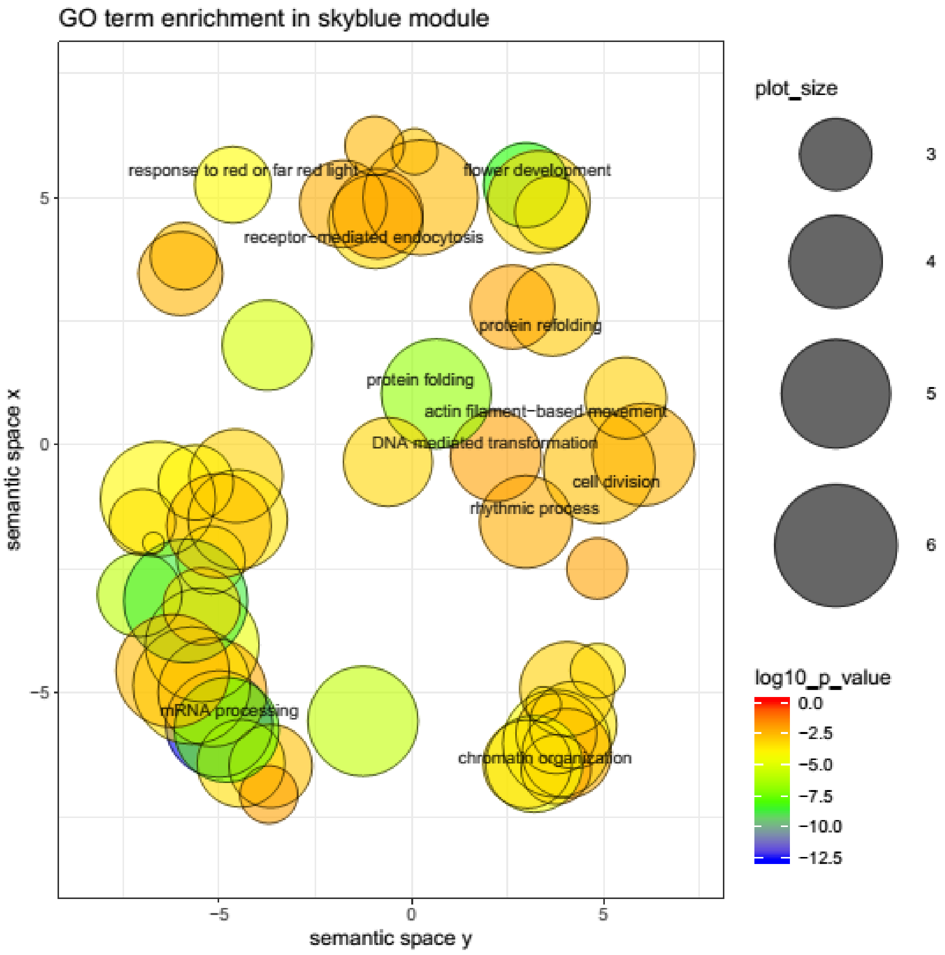

GO term enrichment in skyblue module
response to red or far red light
flower development
receptor−mediated endocytosis
protein refolding
protein folding
actin filament−based movement
DNA mediated transformation
cell division
rhythmic process
mRNA processing
chromatin organization
semantic space x
semantic space y
5
0
−5
−5
0
5
plot_size
3
4
5
6
log10_p_value
0.0
−2.5
−5.0
−7.5
−10.0
−12.5

Fig. S13 KEGG-based functional classification of treatment associated modules. The x-axis indicates the log10 p-value and the y-axis indicates the number of genes on each enriched pathway. The top-10 ranking KEGG pathways of black (a), lightcyan (b), lightgreen (c), magenta (d), orange (e), pink (f), purple (g) and skyblue (h) modules are shown as bar plots.

(a)

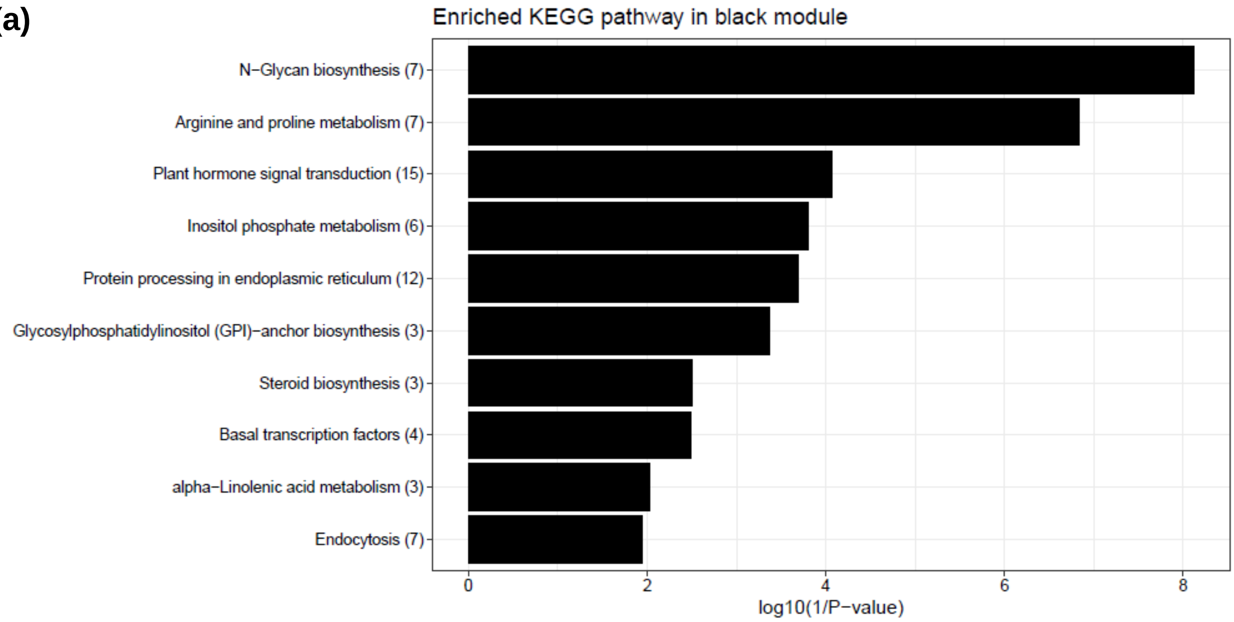

(b)

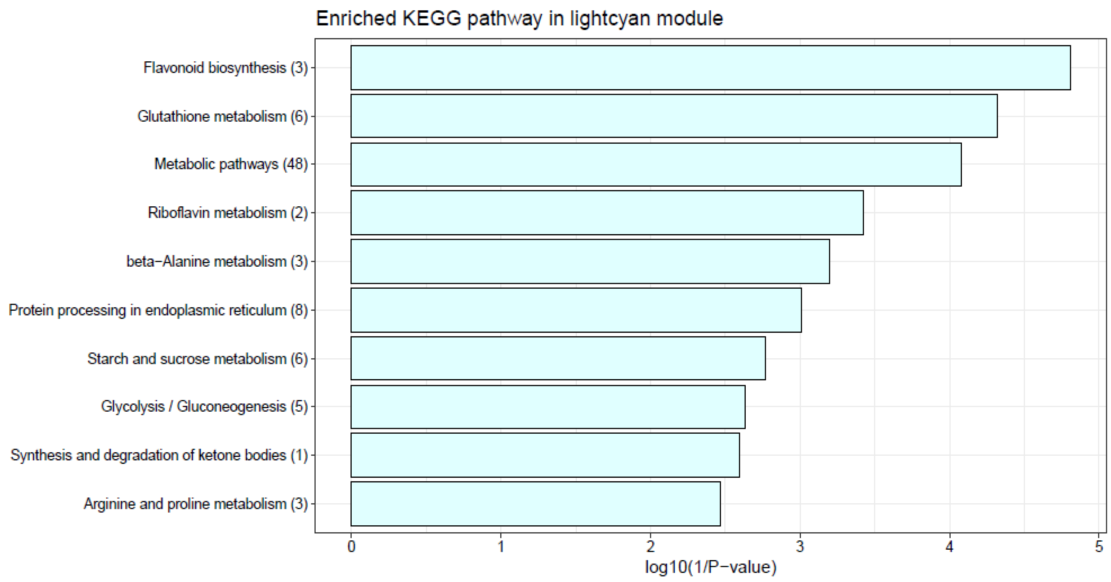
Enriched KEGG pathway in lightcyan module
Flavonoid biosynthesis (3)
Glutathione metabolism (6)
Metabolic pathways (48)
Riboflavin metabolism (2)
beta−Alanine metabolism (3)
Protein processing in endoplasmic reticulum (8)
Starch and sucrose metabolism (6)
Glycolysis / Gluconeogenesis (5)
Synthesis and degradation of ketone bodies (1)
Arginine and proline metabolism (3)
0
1
2
3
4
5
log10(1/P−value)

(c)

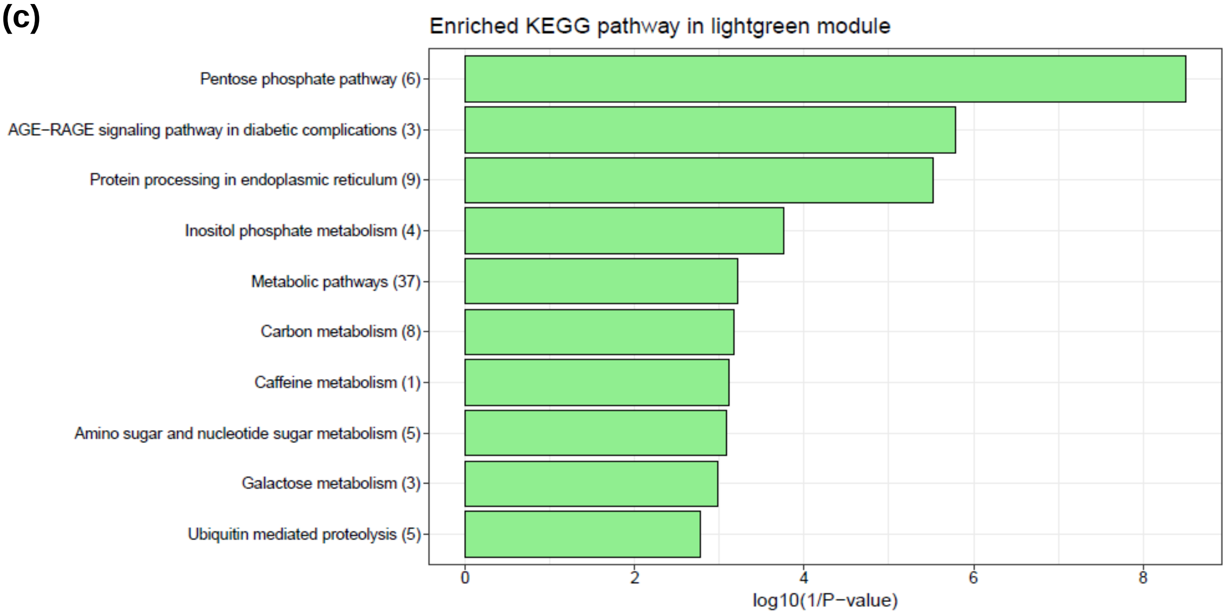
Enriched KEGG pathway in lightgreen module
Pentose phosphate pathway (6)
AGE−RAGE signaling pathway in diabetic complications (3)
Protein processing in endoplasmic reticulum (9)
Inositol phosphate metabolism (4)
Metabolic pathways (37)
Carbon metabolism (8)
Caffeine metabolism (1)
Amino sugar and nucleotide sugar metabolism (5)
Galactose metabolism (3)
Ubiquitin mediated proteolysis (5)
0
2
4
6
8
log10(1/P−value)

(d)

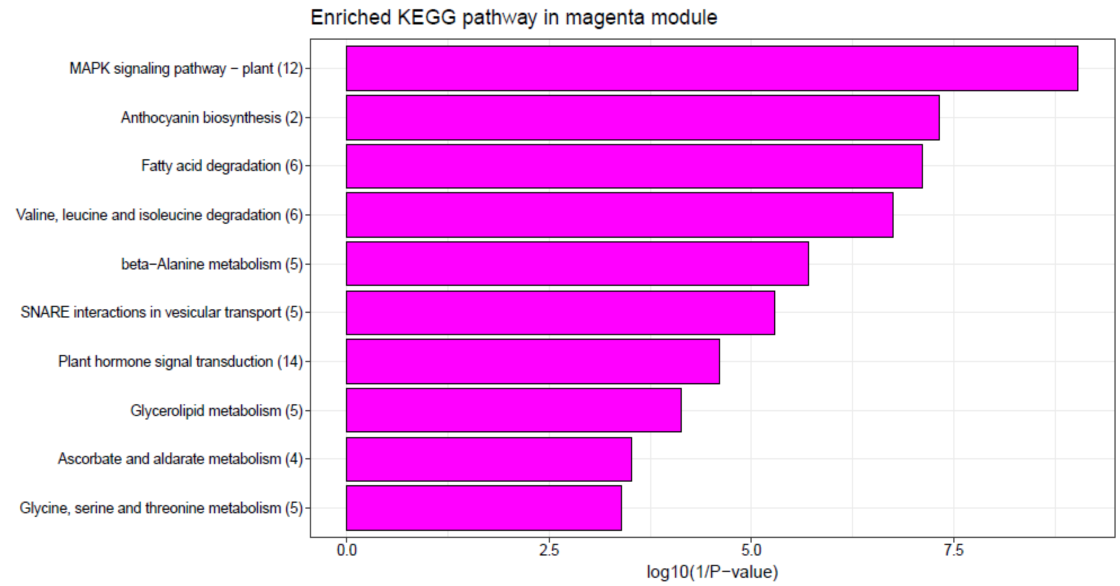

Enriched KEGG pathway in magenta module
MAPK signaling pathway − plant (12)
Anthocyanin biosynthesis (2)
Fatty acid degradation (6)
Valine, leucine and isoleucine degradation (6)
beta−Alanine metabolism (5)
SNARE interactions in vesicular transport (5)
Plant hormone signal transduction (14)
Glycerolipid metabolism (5)
Ascorbate and aldarate metabolism (4)
Glycine, serine and threonine metabolism (5)
0.0
2.5
5.0
7.5
log10(1/P−value)

(e)

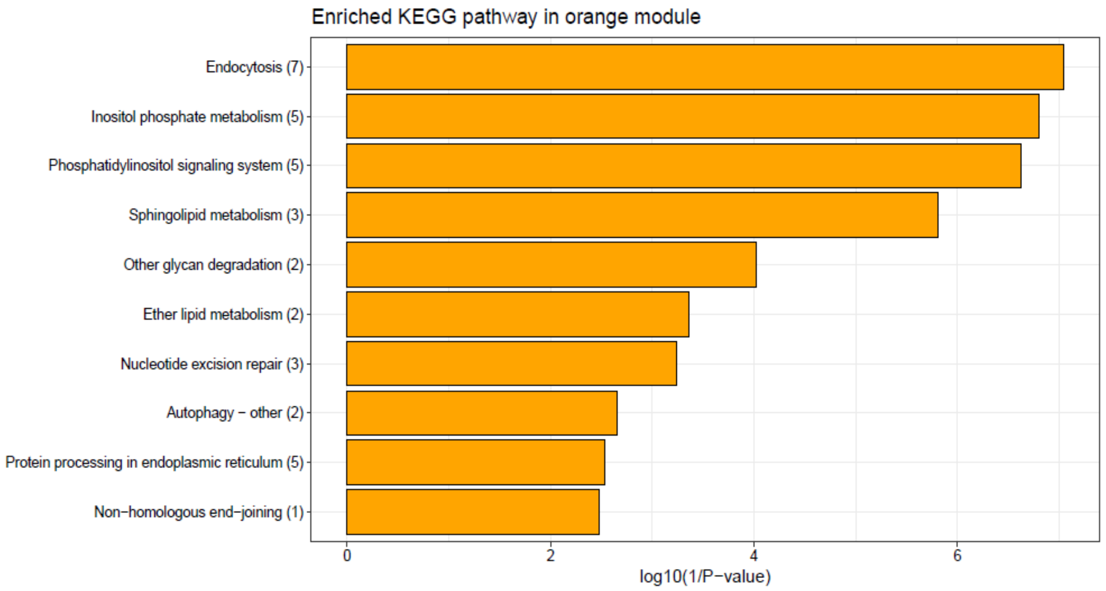

Enriched KEGG pathway in orange module
Endocytosis (7)
Inositol phosphate metabolism (5)
Phosphatidylinositol signaling system (5)
Sphingolipid metabolism (3)
Other glycan degradation (2)
Ether lipid metabolism (2)
Nucleotide excision repair (3)
Autophagy − other (2)
Protein processing in endoplasmic reticulum (5)
Non−homologous end−joining (1)
0
2
4
6
log10(1/P−value)

(f)

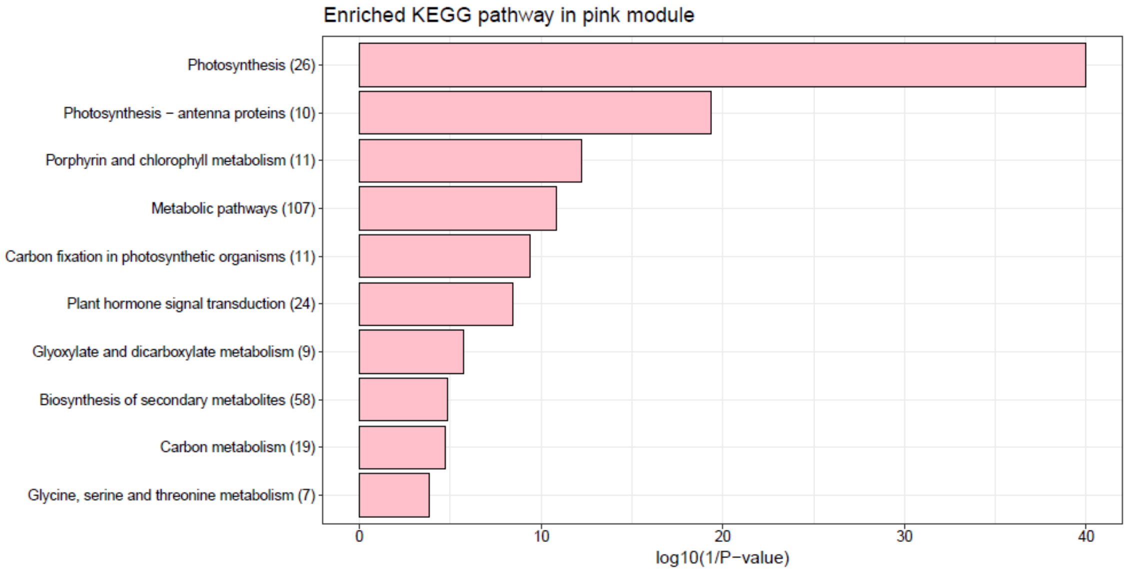
Enriched KEGG pathway in pink module
Photosynthesis (26)
Photosynthesis − antenna proteins (10)
Porphyrin and chlorophyll metabolism (11)
Metabolic pathways (107)
Carbon fixation in photosynthetic organisms (11)
Plant hormone signal transduction (24)
Glyoxylate and dicarboxylate metabolism (9)
Biosynthesis of secondary metabolites (58)
Carbon metabolism (19)
Glycine, serine and threonine metabolism (7)
0
10
20
30
40
log10(1/P−value)

(g)

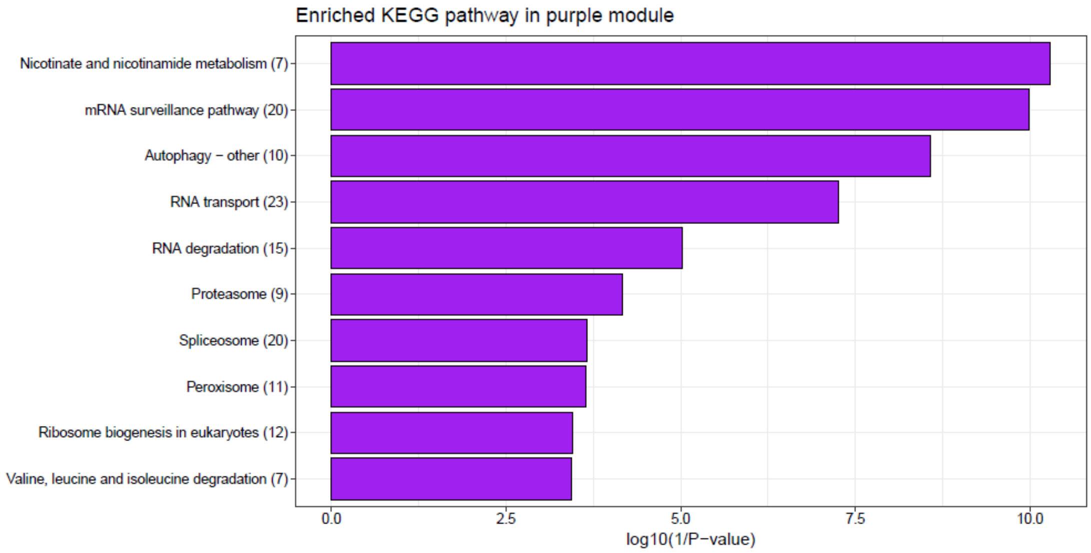
Enriched KEGG pathway in purple module
Nicotinate and nicotinamide metabolism (7)
mRNA surveillance pathway (20)
Autophagy − other (10)
RNA transport (23)
RNA degradation (15)
Proteasome (9)
Spliceosome (20)
Peroxisome (11)
Ribosome biogenesis in eukaryotes (12)
Valine, leucine and isoleucine degradation (7)
0.0
2.5
5.0
7.5
10.0
log10(1/P−value)

(h)

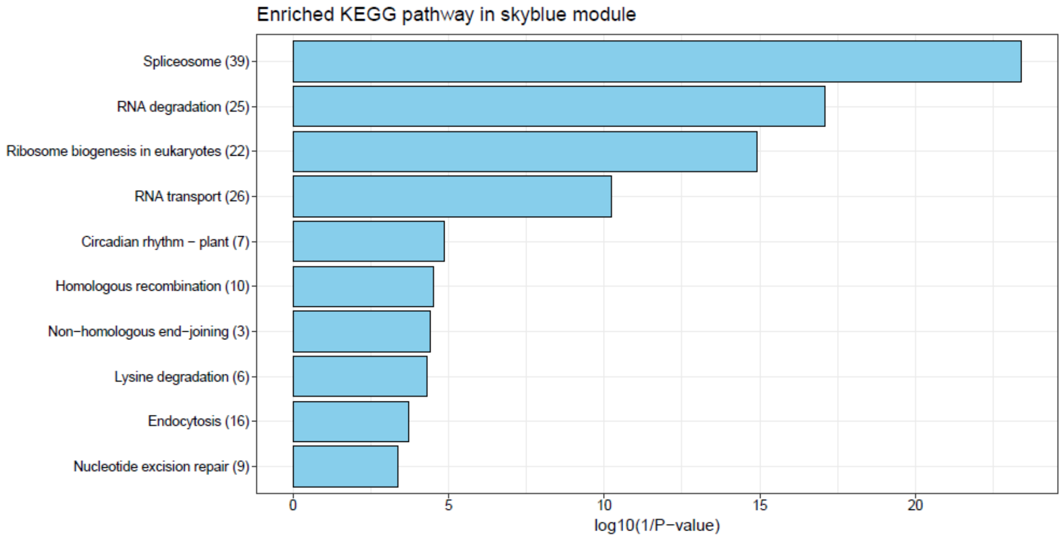

Enriched KEGG pathway in skyblue module
Spliceosome (39)
RNA degradation (25)
Ribosome biogenesis in eukaryotes (22)
RNA transport (26)
Circadian rhythm − plant (7)
Homologous recombination (10)
Non−homologous end−joining (3)
Lysine degradation (6)
Endocytosis (16)
Nucleotide excision repair (9)
0
5
10
15
20
log10(1/P−value)
